# Catalytic filtration: efficient C-C cross-coupling using Pd^(II)^-salen complex-embedded cellulose filter paper as a portable catalyst†

**DOI:** 10.1039/d2ra03440a

**Published:** 2022-07-12

**Authors:** Indah Raya, Svetlana Danshina, Abduladheem Turki Jalil, Wanich Suksatan, Mustafa Z. Mahmoud, Ali B. Roomi, Yasser Fakri Mustafa, Milad Kazemnejadi

**Affiliations:** Department of Chemistry, Faculty of Mathematics and Natural Science, Hasanuddin University Makassar 90245 South Sulawesi Indonesia; Sechenov First Moscow State Medical University Moscow Russia; Medical Laboratories Techniques Department, Al-Mustaqbal University College Babylon Hilla 51001 Iraq abedalazeem799@gmail.com; Faculty of Nursing, HRH Princess Chulabhorn College of Medical Science, Chulabhorn Royal Academy Bangkok Thailand; Department of Radiology and Medical Imaging, College of Applied Medical Sciences, Prince Sattam Bin Abdulaziz University Al-Kharj 11942 Saudi Arabia; Faculty of Health, University of Canberra Canberra ACT Australia; Ministry of Education, Directorate of Education Thi-Qar Thi-Qar Iraq; Biochemistry and Biological Engineering Research Group, Scientific Research Center, Al-Ayen University Thi-Qar 64001 Iraq; Department of Pharmaceutical Chemistry, College of Pharmacy, University of Mosul Mosul Iraq; Department of Chemistry, Faculty of Science, Golestan University Gorgan Iran miladkazemnejad@yahoo.com

## Abstract

A new approach has been developed for environmentally friendly C-C cross-coupling reactions using bi-functional Pd(ii)-salen complex-embedded cellulose filter paper (FP@Si-Pd^II^-Salen-[IM]OH). A Pd(ii)-salen complex bearing imidazolium [OH]^−^moieties was covalently embedded into a plain filter paper, then used as an efficient portable catalyst for the Heck, Suzuki, and Sonogashira cross-coupling reactions under environmentally friendly conditions *via* the filtration method. The catalytic filter paper properties were studied by EDX, XPS, TGA, ATR, XRD, and FESEM analyses. The reactions were catalyzed during reactants' filtration over the catalytic filter paper. The modified filter paper was set up over a funnel and the reactants were passed through the catalytic filter paper several times. The effect of reaction parameters including loading of Pd(ii)-salen complex, temperature, solvent, and contact time were carefully studied and also the optimal model of conditions was presented by the design expert software. High to excellent yields were obtained for all C–C coupling types with 5 to 8 filtration times. Under optimal conditions, all coupling reactions showed high selectivity and efficiency. Another advantage of the modified filter paper was its stability and reusability for several times with preservation of catalytic activity and swellability.

## Abstract 2

A new platform has been developed for environmentally friendly C–C cross-coupling reactions *via* filtration of reactants through a portable Pd^(II)^-salen complex-embedded filter paper.

## Graphical abstract

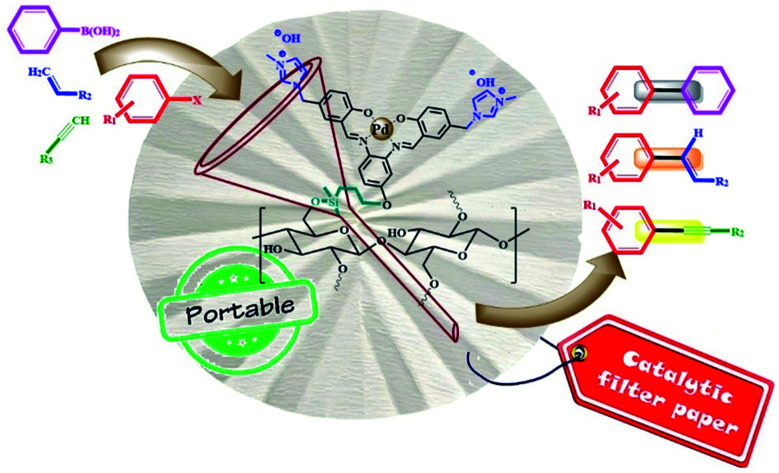

## Introduction

One of the main concerns of researchers is to develop and replace methods for the preparation of organic compounds, which mostly include the non-production of waste materials and consequently the preservation of the environment. From this point of view, heterogeneous and recoverable catalysts have a special place.^1^ The use of filter paper is a smart strategy in organic synthesis for solid phase synthesis which benefits from clean work-up.^2^ Because of the high ability to modify (functionalize) and manipulate a cellulose paper, it has attracted the attention of many scientists in different fields of sciences to achieve various purposes.^3^ In organic chemistry, chemical modifications of cellulose can convert a cellulose paper to a catalyst and could be utilized as a suitable substrate. Previously, Koga *et al.* reported the application of a methacryloxy-modified porous microstructure-cellulose paper as a support for lipase immobilization. The obtained paper showed significant catalytic activity to the non-aqueous transesterification.^4^ Filter paper containing silver nanoparticles was synthesized and utilized by Mourya *et al.* as a catalyst for methyl orange decomposition, cascade reaction, and reduction of nitroarenes. This silver nanoparticle-embedded filter paper was synthesized by immersing FP in hydrophobic AgNO_3_ monodispersed solution.^5^ Cellulose paper is also a good substrate for the NP preparation *in situ*, which was developed for Co^6^ or Ni^7,8^ NPs.

In another work, cellulose filter paper modified with 3-mercapto-propanoic acid was used as an adsorbent to efficiently remove of arsenate from drinking water.^9^ Wei and colleagues introduced a Fe-tannin- framework ink coating for the preparation of cellulose-based catalysts by cellulose filter paper. The obtained Fe_3_C/Fe–N–C catalysts were utilized to reduce oxygen under alkaline conditions.^10^ In addition, the filter paper loaded with gold nanoparticles shows a high level of Raman efficiency (SERS) that supply real-time monitoring for chemical reactions.^11^ Ag-doped cellulose FP as an antibacterial wound dressing,^12^ synthesis of cellulose paper with superhydrophobic property for water drop energy harvesting,^3^ paper-based electrodes,^11^ and self-cleaning superhydrophobic cellulose paper,^13^ were some of the recent advances in the field of cellulosic filter paper manipulation, which reflects its high potential for application in various fields of science.

The results well show that the surface modification of a cellulose paper with nanoparticles as well as organic compounds is possible through covalent bonding or electrostatic interactions. Despite some advantages associated with the heterogeneous catalysts such as recoverability and ease of operation (work-up), their catalytic activity is decrease during consecutive recycles due to metal leaching, lack of stability in the mixture, and poisoning of the active sites.^1^ Contamination with the reactants also requires tedious and frequent washing and defective recycling from the reaction mixture were another disadvantages of heterogeneous catalytic systems which reduces its activity. The design of a catalytic system based on a portable catalytic filter paper with a filtration protocol eliminates the above-mentioned drawbacks and directs the reactions to green conditions. Although various studies have been performed on modified cellulose filter paper for its catalytic applications, their activity has not been studied from the point of view of catalyzing reactions through the sequential filtration of raw materials as a portable catalyst, and in all cases, the modified filter paper, has been used as an immersion (like a traditional heterogeneous reaction).

The undeniable importance of coupling reactions such as Stille, Heck, Suzuki, Sonogashira, *etc.* in various fields from the preparation of important pharmaceutical compounds to agriculture and the chemical and petrochemical industries, has led researchers to seek new cost-effective methods, with high efficiency as well as easy work-up to use in the industry and larger scales. Pd-based catalytic systems are one of the most reliable methods for synthesizing these couplings, which have been used in various forms of (1) solid-supported metal ligand complexes, (2) discrete soluble palladium complexes, (3) soluble Pd NPs, (4) supported Pd nano- and macroparticles, (5) palladium-exchanged oxides, and (6) soluble ligand-free Pd so far.^14^ On the other hand, research has shown that imidazolium tails bearing hydroxide counter ions in the heterogeneous catalytic systems have a high basicity towards coupling reactions (as a basic reagent), so that their presence in the catalyst structure in the coupling reactions causes the reaction to take place in the absence of any basic reagent.^15–18^

**Scheme 1:**
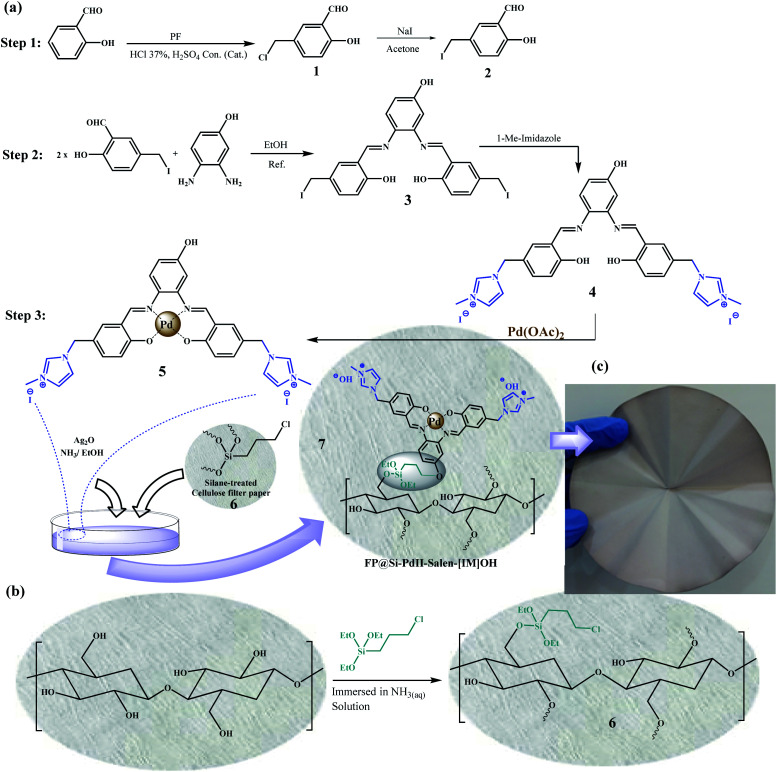
The synthesis of (a) Pd(ii)-salen-[IM]I complex (5) and (FP@Si-Pd^II^-Salen-[IM]OH) (7), and (b) silylation of the cellulose filter paper by CPTES (FP@Si-Cl). (c) Original picture of the prepared FP@Si-Pd^II^-Salen-[IM]OH filter paper.

In this way, in the present work, a modified cellulose filter paper was prepared by immobilization of a Pd(ii) salen complex bearing imidazolium hydroxide tails on a silica treated cellulose filter paper, as a portable catalytic system (Scheme 1). Scheme 1c shows an original picture of the resulting cellulose filter paper after surface modification. The application of this system was recently demonstrated by the transfer hydrogenation of nitroarenes using cellulose filter paper-supported Pd/C by filtration methods.^19^

**Scheme 2:**
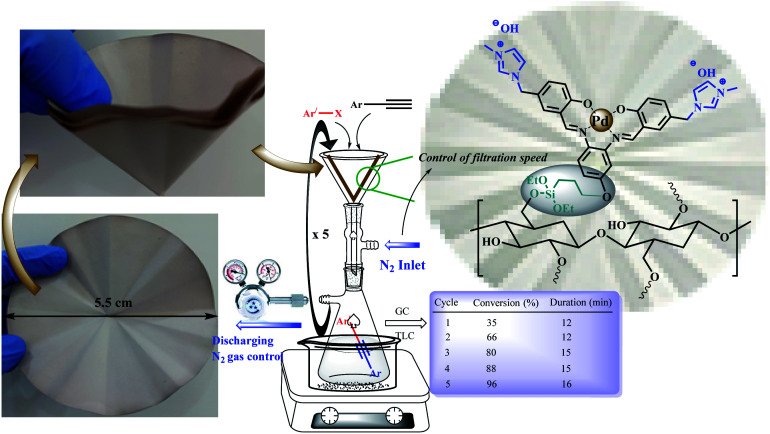
A schematic set up for the FP@Si-Pd^II^-Salen-[IM]OH catalyzed Sonogashira cross-coupling reaction. The left images show the original pictures of the prepared FP@Si-Pd^II^-Salen-[IM]OH filter paper.

The catalytic activity of the modified cellulose paper was studied on Heck, Sonogashira, and Suzuki cross-coupling reactions in depth (Scheme 2), which are among the key reactions used in organic synthesis.^1^ Different reactions could be the subject of this study, but due to the importance of such reactions and the need for a stable and available catalyst, these reactions were selected. On the other hand, due to the involvement of several parameters in this protocol, the coupling reactions were selected so that their product is soluble in the reaction medium and does not deposit on the filter paper.

In this method, one of the most challenging problems in the synthesis of organic compounds, namely “effective concentration”, was overcome. In common methods for the synthesis of organic compounds, as the reaction progresses, the concentration of the raw materials decreases and the effective collisions of the raw materials with each other as well as with the catalyst surface (in the case of heterogeneous catalysts) are reduced and stop the reaction progresses. But with the design of a catalytic filter paper, the catalytic process is subject to the passage (filtration) of raw materials and forced contact with the catalyst surface. Thus, the reaction takes place only on the part of the material that is being filtered, causing the reaction to proceed to the completion.

## Results and discussion

### Characterization of 2,3,4,5 compounds

Characterization studies were performed in two phases on compound 5 as well as on filter paper 7 step by step and after confirming formation of the desired product in each step, the synthesis continued and the compound was used for the next step. Fig. S1,S3–S10† shows the results of FTIR and NMR (^1^H & ^13^C) spectroscopy for 1-5. The presence of characteristic peaks at 674 cm^−1^ and 2856 cm^−1^, respectively, related to the stretching vibrations of C–Cl and C–H bonds (aliphatic, belonging to the methylene group) confirms the structure of 5-chloromethyl salicylaldehyde (1) in full agreement with the previous reports (Fig. S1a†).^20–22^ The peak corresponding to the stretching vibration of C=O (aldehyde group) also appeared at 1660 cm^−1^. Fig. S1b† shows 5-iodomethyl salicylaldehyde (2) FTIR spectrum, in which the peak appearing at 610 cm^−1^ (weaker stretching vibration than C–Cl)^23^ was attributed to C–I stretching vibration. Stretching vibrations related to C–H (aliphatic) and C=O (aldehyde group) also appeared at 2841 cm^−1^ and 1663 cm^−1^, respectively (Fig. S1b†), in agreement with the literature.^24,25^ The salen ligand (3) was characterized by the shift of the peak belonging to the aldehyde group towards weaker vibrations due to the formation of C=N bond at 1635 cm^−1^.^25,26^ Also, the peaks related to C–I and C–H (aliphatic) and C–H (aromatic) have appeared at 582 cm^−1^, 3078 cm^−1^ and 2916 cm^−1^, respectively (Fig. S1c†).^23,24,26^ Substitution of methyl imidazolium groups has caused a series of stretching vibrations related to the C–H of the imidazole ring at 1500–2950 cm^−1^ in agreement with the literature (Fig. S1d†).^15,16,22^ The peaks that appeared at 1625 cm^−1^ and 1543 cm^−1^ were also attributed to the stretching vibrations of the amidine (–N–C=N) and C=C groups in the imidazole ring.^15,16,22^ The peak at 1650 cm^−1^ also shows the stretching vibration of C=N bond with strong intensity. Pd coordination to the salen ligand shifted the C=N stretching vibration from 1650 cm^−1^ to the weaker 1620 cm^−1^ wavenumbers, which was strong evidence for the formation of the Pd^II^-salen complex (Fig. S1e†).^25,27^ In addition, the two peaks at 462 cm^−1^ and 568 cm^−1^, respectively, related to the stretching vibrations of Pd–O and Pd–N bonds, confirm the coordination of Pd to the salen ligand.^25,27^ Also, the intensity of stretching vibration related to O–H (phenolic) was significantly reduced due to this coordination. It is also worth mentioning that based on previously published articles, different synthetic pathways were evaluated to prepare compound 5 as well as filter paper 7, and the synthetic pathway shown in the Scheme 1 was the most optimal and reproducible pathway in order to prepare the catalytic filter paper.

### Filter paper characterization

Catalytic filter paper 7 was characterized analytically and spectroscopically step by step. In first, the papers were studied by ATR-IR analysis (ESI, Fig. S2†). In comparison with the ATR-IR spectrum of the pristine cellulose paper (Fig. S2a†), ATR-IR spectrum of the silylated paper confirmed the silylation by the presence of the vibrations at 1160 cm^−1^ (strong) and 805 cm^−1^ (weak) attributed to the stretching vibrations of asymmetric and symmetric Si–O–C, respectively (Fig. S2b†).^28^ Also, a peak appeared at 1518 cm^−1^ shows the stretching vibration related to Si–C.^29,30^ The stretching vibration related to C–Cl bond at 636 cm^−1^ with strong intensity was another poof for the successful silylation of FP.^29,30^ The characteristic peaks correspond to the imine and olefin bonds at 1645 cm^−1^ and (1442–1643) cm^−1^, respectively, represent the successful immobilization of complex 5 on the silylated cellulose paper (Fig. S2c†). Also, two peaks appeared at 534 cm^−1^ and 558 cm^−1^ show the stretching vibrations related to Pd–O and Pd–N bonds. EDX and ICP analyses confirmed the successful functionalization of the plain cellulose filter paper, wherein % wt Si was found to be 14.68, and 11.36 respectively.^19^ This amount provides suitable substrate for the next functionalization on the silylated sites.

**Fig. 1:**
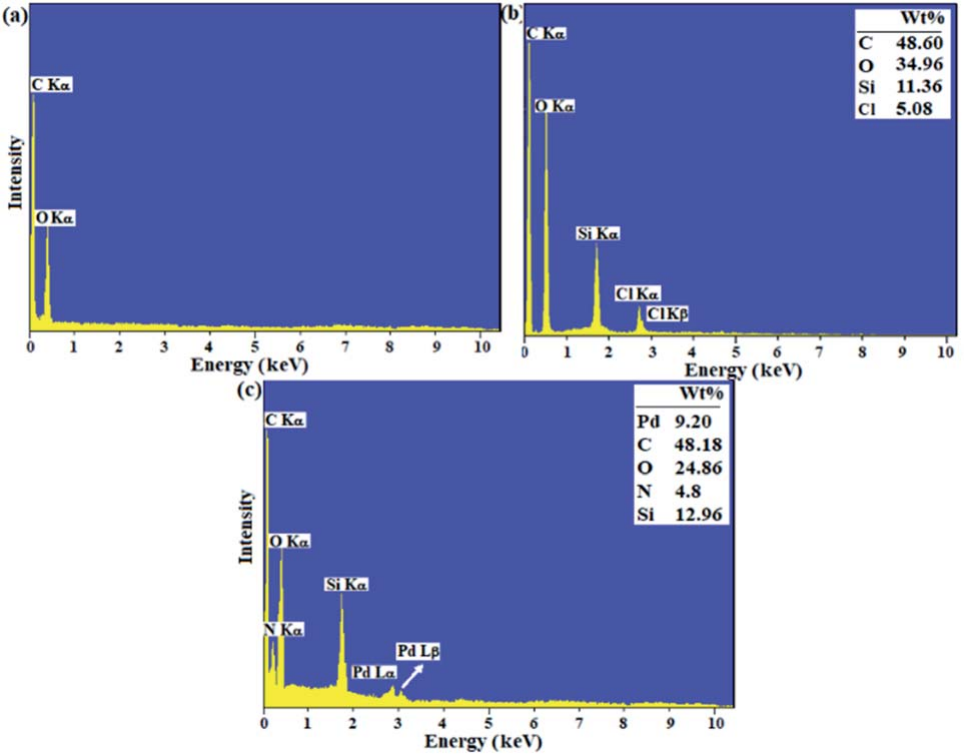
EDX spectra of (a) plain cellulose paper, (b) SiCFP, and (c) FP@Si-Pd^II^-Salen-[IM]OH.

The loading contents of Si and Pd were measured on the prepared filter papers 6 and 7, respectively (Scheme 1) by ICP-MS analysis of the resulting ash from the filter paper. For this purpose, the modified filter paper was placed in a crucible (0.9 g) and then calcinated in an oven at 500 °C under air atmosphere. At the same time, an unmodified (pristine) filter paper was calcinated as a control, under exactly the same conditions. Based on the results of ICP-MS analysis, the modified filter paper 7 contains 9.45 wt% Pd and 13.2 wt% Si. The elemental composition of filter papers 6 and 7 (catalytic filter paper) was also studied by EDX analysis. Fig. 1a shows the FTIR spectrum of the plain filter paper with the detection of only C, O elements that reflects its purity. Two peaks at 2.6 and 2.8 eV binding energies in the EDX spectrum of SiCFP (Fig. 1b) were assigned to Cl *K*α and Cl *K*β respectively, demonstrating successful modification of the filter paper with CPTES.

The identification of Pd and N elements in the modified filter paper with Pd^II^-salen complex 5 at binding energies of (2.9 and 3.1 eV) and 0.3 eV, respectively, proves the successful immobilization of the complex on the FP. Complete removal of peaks related to Cl-related binding energies indicates that all silica groups were involved in the immobilization of the Pd^II^-salen complex and also the immobilization of the complex occurred through covalent bonding according to what is shown in Scheme 1 (Fig. 1c).

**Fig. 2:**
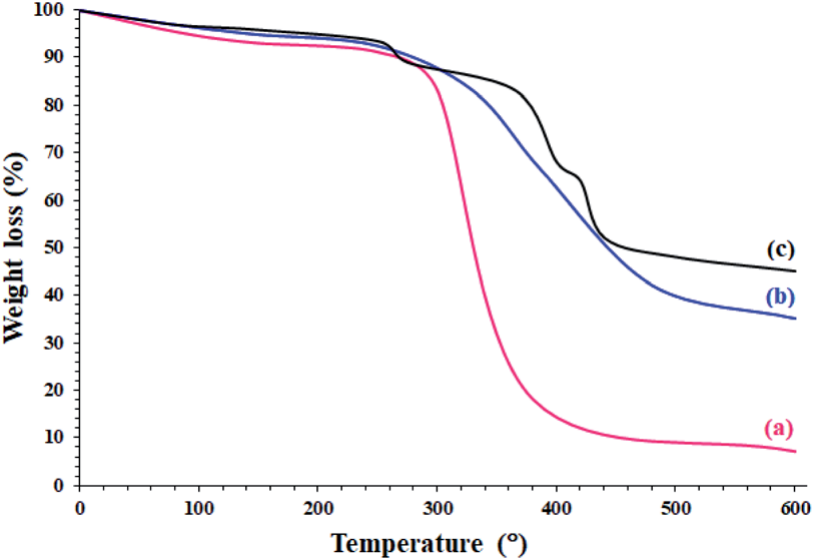
TGA analyses of (a) unmodified (pristine), (b) silylated, and (c) FP@Si-Pd^II^-Salen-[IM]OH filter papers.

The study on the thermal behavior of the modified and unmodified papers clearly confirmed the silylation and functionalization of the filter paper with the Pd^II^-salen complex. Decomposition of the unmodified filter paper takes place in one step, starting at 280 °C and ending with a steep slope at 410 °C (Fig. 2a).^31^ Silylation of the filter paper causes a significant increase in the thermal stability of the filter paper. As shown in Fig. 2b, the thermal decomposition was performed with a gentle slope and at the end, the residual weight was about 35%, which by abstraction from the remaining weight of the filter paper in the previous step, the residual weight of 28% could be attributed to the remaining silica groups. This thermal behavior is similar to the thermal behavior of the previously reported crosslinked polymers.^32^ The presence of silica groups on cellulose fibrous causes Si–O–Si bonds formation and consequently reduces the mobility of cellulose fibrous and provides high thermal stability of the filter paper.

Immobilization of the Pd^II^-salen complex due to the presence of C=N bonds has given more stability than the silylated filter paper (Fig. 2c). Schiff base compounds have a special place in heat-resistant polymers,^33^ therefore, the immobilization of the Pd-salen complex (with coordinated Pd ions) has resulted in greater thermal stability in the catalytic filter paper. The presence of three peaks in the thermal decomposition of FP@Si-Pd^II^-Salen-[IM]OH was evidence of the presence of multiple immobilized phases on the surface of filter paper, which was in agreement with previous analyzes. As shown in Fig. 2c, two peaks appearing at 275 °C (corresponding to 13% weight loss) and 366 °C (corresponding to 3% weight loss) could be attributed to the decomposition of the imidazolium moieties (according to their weight loss percentages) and the Pd^II^-salen ligand decomposition, respectively. The final peak at 420 °C was also related to the residual decomposition of silica-functionalized cellulose fibrous.

**Fig. 3:**
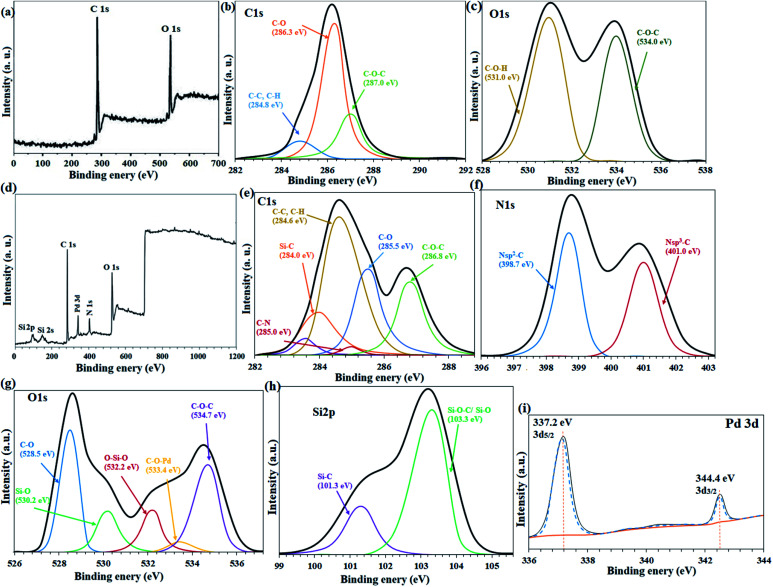
(a) Overall survey, and deconvulated high resolution (b) C 1s, and (c) O 1s XPS spectra of the plain cellulose paper. (d) Overall survey, and deconvulated high resolution (e) C 1s, (f) N 1s, (g) O 1s, (h) Si 2p, (i) Pd 3d XPS analyses of FP@Si-Pd^II^-Salen-[IM]OH filter paper.

Fig. 3a shows the XPS overall survey analysis (full range) of the unmodified filter paper with the presence of C and O elements, in agreement with the corresponding EDX analysis. Also, C 1s (Fig. 3b) and O 1s (Fig. 3c) deconvolution spectra showed the atomic center of the related functional groups for the cellulose filter paper.^34,35^ The C 1s spectrum for the unmodified cellulose corresponds to three types of carbon atoms that can be attributed to (C–C, C–H), C–O and C–O–C bonds at 284.8 eV, 286.3 eV and 287 eV binding energies, respectively (Fig. 3b).^34^ In full agreement with the previous reports,^34,36^ oxygen-containing bonds such as C–O and C–O–C have higher intensities than C–C, due to the high content of these bonds in the cellulose structure. The O 1s spectrum was consistent with the presence of two types of oxygen atom as C–O–H and C–O–C bonds at 531 eV and 534 eV, respectively, in agreement with the structure of the fibrous cellulose (Fig. 3c).^37^

The presence of the expected elements in FP@Si-Pd^II^-Salen-[IM]OH filter paper was confirmed by the overall survey XPS analysis (full range) of the filter paper including C, N, O, Si, and Pd elements (Fig. 3d). Also, the deconvulated high resolution C 1s, N 1s, O 1s, Si 2p, and Pd 3d spectra gave useful information about functional groups and elemental composition for FP@Si-Pd^II^-Salen-[IM]OH filter paper (Fig. 3e–i). The C 1s spectrum for FP@Si-Pd^II^-Salen-[IM]OH corresponds to six different types of bonds for carbon, which in comparison with the C 1s spectrum for the unmodified filter paper (Fig. 3b), indicates the successful silylation and immobilization of the Pd-salen complex on the filter paper.^38^ As shown in Fig. 3e, a peak at 284 eV, related to Si–C bond in C 1s spectrum of the filter paper, confirms the successful immobilization of CPTES groups on the filter paper.^39^ Also, two peaks at 283.6 eV and 285 eV for C–N and C=C bonds, respectively, confirmed the covalent immobilization of Pd-salen complex on the filter paper.^39,40^ The 284.6 eV, 285.5 eV, and 286.8 eV binding energies can also be attributed to the C–C/C–H, C–O, and C–O–C bonds, respectively.^39,40^

The two characteristic peaks at 398.7 eV and 401.0 eV were attributed to Nsp^2^–C and Nsp^3^–C bonds in the N 1s deconvulated spectrum, respectively (Fig. 3f).^38^ The Nsp^2^–C peak indicates the presence of C–N=C imidazole groups in the filter paper framework.^39^ In addition, the Nsp^2^–C bond was also consistent with the ionic moiety of the imidazole rings.^41^ As shown in O 1s high resolution region (deconvulated), five different oxygen-related functional groups were detected in the spectrum (Fig. 3g). Two characteristic peaks at 530.0 eV and 532.2 eV shows the binding energies related to Si–O and O–Si–O bonds in agreement with the previously reported XPS O 1s results for the silylated cellulose fibers (Fig. 3g).^30,42^ Also, the coordinated Pd to O atom (C–O–Pd in the Pd-salen complex) was appeared with a low intensity at 533.4 eV (Fig. 3g).^40^ Two other high intensity peaks were assigned to C–O and C–O–C bonds at 528.5 and 534.7 eV, respectively (Fig. 3g).^35,36,42^ Cellulose filter paper silylation through Si–C and Si–O–C/Si–O bonds was confirmed by the presence of 101.3 eV and 103.3 eV binding energies in the high-resolution XPS Si 2p spectrum, respectively (Fig. 3h).^43^ High resolution XPS Pd 3d analysis determined the oxidation state of the coordinated Pd in the salen ligand immobilized on the cellulose filter paper (Fig. 3i). As shown in Fig. 3i, the Pd 3d_5/2_ and Pd 3d_3/2_ related peaks have appeared at 337.2 eV and 344.4 eV binding energies, respectively, which have an energy band gap of 7.2 eV, exactly belonging to Pd^+2^ (Fig. 3c).^44^

**Fig. 4:**
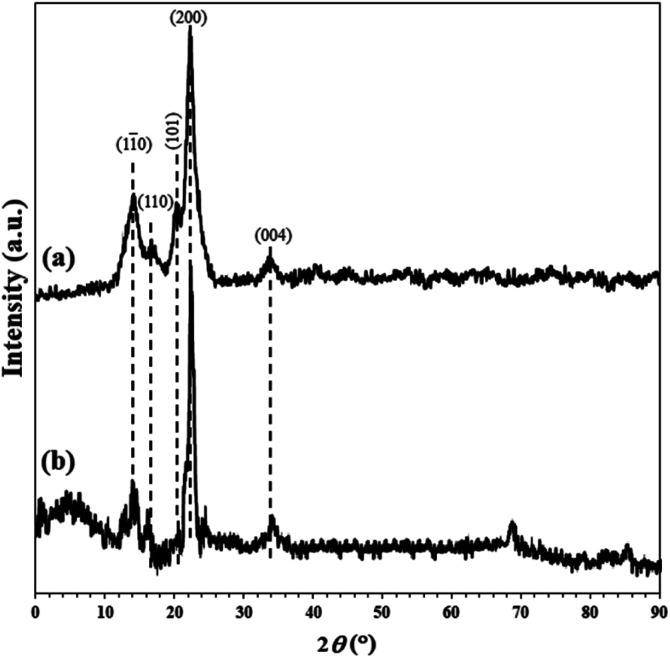
XRD patterns of (a) plain cellulose filter paper and (b) FP@Si-Pd^II^-Salen-[IM]OH.

XRD analysis of the unmodified and FP@Si-Pd^II^-Salen-[IM]OH filter papers were shown in Fig. 4. The presence of peaks appearing at 2*θ* = 14.1°, 16.2°, 20.3°, 22.2°, and 34.1° corresponding to the (1 1̄ 0), (110), (101), (200) and (004) planes respectively, were in complete agreement with the monoclinic cellulose type 1 crystal structure (PDF files: 000561717, 000561718 and 000561719),^45,46^ that confirmed the crystalline structure of the cellulose FP in agreement with the previous reports (Fig. 4a).^6,19^ XRD pattern of the FP 7 confirmed the existence of two different phases, including silicate groups and the Pd complex. According to the filter paper X-ray diffraction pattern of 7, the amorphous peak appearing at 2*θ* = 4.3° corresponds to the amorphous structure of silicate groups on the cellulose fibrous. As shown in Fig. 4b, the immobilization of Pd complex on the cellulose causes the XRD pattern of the cellulose to be deviated from crystalline state in full accordance with the X-ray diffraction patterns reported from immobilized Pd complexes (Fig. 4b).^47^

The surface area and porosity of the modified and plain FPs were evaluated by BET method. The specific surface area and the average pore size of the cellulose FP, FP@Si-Cl, and FP@Si-Pd^II^-Salen-[IM]OH was in agreement with other analyses and modification of the FP in each step. Based on the results, the plain FP has a pore size and a specific surface area of 8.66 microns and 1.52 m^2^ g^−1^ respectively, which reaches to 6.46 microns and 1.66 m^2^ g^−1^ after silylation.^19^ The specific surface area increased to 3.55 m^2^ g^−1^ after surface modification of the silylated FP with Pd-salen complex. Also, the average pore size was decreased to 1.60 microns upon this modification.

**Fig. 5:**
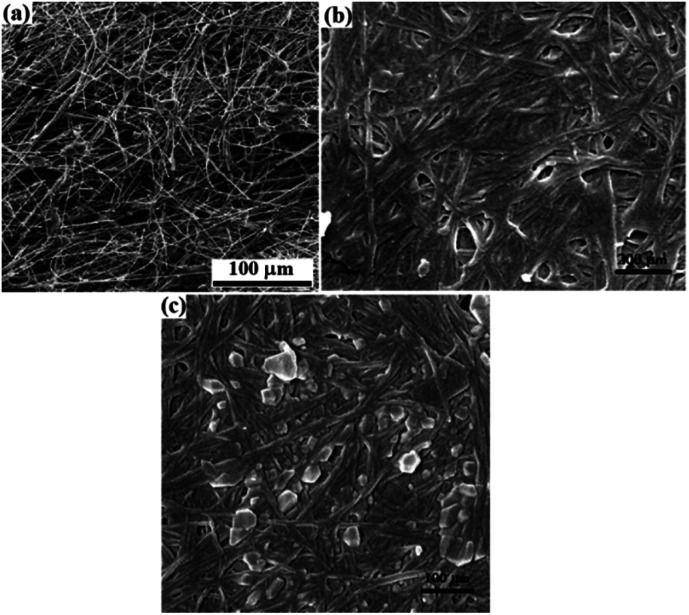
FESEM images of (a) plain FP,^19^ (b) SiCFP,^19^ and (c) FP@Si-Pd^II^-Salen-[IM]OH.

Fig. 5 shows the SEM images taken from the plain and modified papers. FESEM images from the plain and silylated filter paper showed an increase in average diameter of 7 μm (from about 16 μm for the plain filter paper to about 23 μm for the silylated filter paper)^19^ with a decrease in porosity after the silylation of the filter paper, which confirmed the success of the functionalization (Fig. 5a and b). Different plate-shaped morphology for the functionalized filter papers can be attributed to the crosslinking between the silica groups. In addition, the brighter spots in the SEM image of FP@Si-Pd^II^-Salen-[IM]OH was related to the immobilized complex 5 on the silylated filter paper (Fig. 5c).

### Screening of the reaction parameters

Four effective parameters including Pd loading amount, reaction temperature, paper surface area and N_2_ inlet gas pressure were analyzed by Design Expert software and the designs were studied on the reaction of Sonogashira model (coupling of phenylacetylene with iodobenzene). Tables S6† to S8 show the results of some of these tests. Based on these analyzes, Sonogashira efficiency depends on all 4 parameters (ESI, Fig. S11 –S13 and Tables S1–S8†). First, the effect of contact surface duration (filtration time) on the coupling reaction efficiency was studied. Increasing the contact time of the reaction mixture with the filter paper surface placed on the funnel was done by applying low N_2_ pressure into the vacuum Erlenmeyer flask placed under the funnel. Table S1† shows the results of this study in which, by applying 0.3 bar pressure during 5 consecutive filtrations for 70 min, the efficiency reaches 96%. As shown in Table S1,† as the pressure inside the Erlenmeyer decreases, the filtration time also decreases exponentially, resulting in reduced efficiency. In this case, the number of filtration times should be increased, which is not desirable (Table S1,† entries 1 and 2). On the other hand, increasing the pressure inside the Erlenmeyer flask to 0.7 bar did not have a significant effect on reaction efficiency (Table S1,† entries 4 and 5).

It is also important to note that the contact time could be increased by adding another raw filter paper. But more repetitive results were obtained using N_2_ gas flow. On the other hand, filter paper contamination reduces efficiency. Control experiments (will be shown in the next section) showed that the presence of oxygen also plays an inhibitory role in contact with the catalytic filter paper and reduces efficiency.

The loading contents of Pd-salen complex 5 on 95 cm^2^ surface area (on one filter paper with a diameter of 5.5 cm), water bath temperature, and solvent were studied as three effective and vital parameters in the Sonogashira model coupling reaction. It should be noted that due to the basic nature of the filter paper (which will also be shown in control experiments), no basic reagent was used in all reactions. The reaction of iodobenzene with phenylacetylene (Sonogashira coupling) was selected as the model reaction. In the first step, loading contents of Pd-salen complex 5 on the filter paper with an area of 95 cm^2^ was studied based on the Pd content. For this purpose, different concentrations of ethanolic Pd-salen complex solution including 0.3, 0.7, 1.2, 2.5 mmol Pd were prepared and immobilized on the FP based on the procedure described in the experimental section. Table S2† shows the effect of different Pd loading amounts on the FP. The obtained results indicated that at a loading amount of 1.0 mmol of Pd, the highest possible efficiency for sonogashira coupling product, 1,2-diphenylethyne, was equal to 96% obtained during 5 consecutive filtrations (70 minutes) (Table S2,† entry 3). Increasing the loading amount to 2 mmol had no effect on the Sonogashira coupling product, but at 2 mmol of Pd loading, the reaction time increased significantly and the efficiency decreased slightly. On the other hand, at loading amounts of 0.2 and 0.5 mmol of Pd, the efficiencies decreased to 72% and 86%, respectively. Considerably, a significant increase in reaction time was consistent with an increase in Pd content in the filter paper. As the Pd loading on the complex increases, the porosity of the filter paper decreases and the reactants become more difficult to filtrate. Although this reduction in porosity increases the contact time between the filter paper surface and the reactants, this increase in contact time does not lead to a significant increase in efficiency. On the other hand, polar–polar interaction between polar groups of silica (in addition to polar OH groups in cellulose) in the filter paper and the polar solvent EtOH : H_2_O, increases the reaction time, as observed by different solvents.

It seems that the reaction time (including successive filtrations) and consequently the reaction efficiency is affected by the two factors of viscosity and polarity of solvents. In order to use total surface of the filter paper (covering the whole of surface of the FP with the mixture) the amount of solvent was 5 mL for all tests. Table S3† shows the effect of a wide range of solvents with different polarities and viscosities on the preparation of 1,2-diphenylethyne. Water and ethanol gave 40% and 85% efficiencies for 60 and 50 minutes, respectively (a total of 5 consecutive filtrations) (Table S3,† entries 4, 5). But in a 1 : 2 mixture of H_2_O : EtOH, the efficiency reached 96% for 70 min. It seems that the addition of water to the reaction mixture causes better diffusion and interaction of the reactants in the filter paper layers and consequently their stronger interaction with the catalytic active sites. The reaction time of high viscosity solvents such as butyl acetate (2.98 cp) and DMSO (2 cp) than the H_2_O : EtOH mixture, increased to 94 and 70 min, respectively, but still provided lower efficiency than H_2_O : EtOH mixture. No observable products were found in the non-polar solvent of hexane (Table S3,† entry 9). Solvents such as CH_3_CN and DMF also did not give high efficiency (Table S3,† entries 3,2).

To investigate the effect of temperature on the Sonogashira model coupling reaction, the whole set up was performed in a glove-box (air atmosphere). The reaction was placed on a water bath with adjustable temperature. According to the optimal performance of EtOH : H_2_O, the reaction was studied at three different temperatures of 27, 50 and 70 °C. The results showed that the temperature was the least effective parameter (relative to the Pd content in the filter paper and solvent) in the Sonogashira coupling reaction. As shown in Table S4,† increasing the reaction temperature to 70 °C only reduced the reaction time by 7 minutes. Therefore, the coupling reactions were performed at an optimum temperature of 50 °C (on a water bath) and in EtOH : H_2_O solvent on FP@Si-Pd^II^-Salen-[IM]OH with a thickness and diameter of 0.2 mm and 5.5 cm, respectively, containing 1.0 mmol of Pd.

Scheme 1 shows the steps of preparing the catalytic filter paper schematically. It should be noted that to prepare FP@Si-Pd^II^-Salen-[IM]OH, different synthetic pathways were examined and tested, and finally the synthetic pathway shown in Scheme 1 was used as a reliable and reproducible route. As shown in Scheme 1, in order to avoid the concerns related to metal leaching during the various stages of catalyst preparation, Pd coordination to the salen ligand takes place in the final step.

The effect of surface area was studied by cutting filter paper with a diameter of 5.5 cm into square pieces with a length of 10 mm. For this purpose, the set up reaction was changed and the reaction was performed in the presence of cutted pieces of the filter paper and stirred by a magnet.

According to the results presented in Table S5,† the surface area (which is another reflection of the loading amount of Pd complex) has a significant effect on the reaction efficiency in full agreement with the loading effect of Pd (Table S5†); because it reduces the surface area corresponds to a decrease in the amount of catalytically active sites. However, in this set up, a significant difference was observed compared to the filtration set up.

As shown in Table S5,† the highest efficiency occurs in this set up at 95 cm^2^ of the filter paper, which was equal to 80% for 70 minutes (50 °C). One of the main reasons could be the sticking of the pieces of the filter paper to each other as well as the wall of the reaction balloon, and consequently the reduction of improper interaction between the reactants and the surface of the filter paper; In other words, homogeneous interaction is not achieved. The phenomenon of mass transfer in the filtration method also acts as a driving force that catalyzes the reaction while passing through the filter paper (filtration). Effective concentration was another important factor that probably affects the high efficiency observed for the filtration method. With the diffusion of the reactants to the filter paper, the effective concentration in this volume increases and leads to the achievement of maximum efficiency. This phenomenon has also been observed for cross-linked catalysts.^48–51^ In other words, in the filtration method, only a part of the reaction mixture in contact with the catalyst surface intends to pass through the catalyst, and this issue increases the effective concentration at the catalyst surface and increases the efficiency. This experiment also showed that the filtration method, as a new and portable method, has an advantage over the heterogeneous method (cutted paper).

Functionalization of the filter paper with Pd^II^-salen complex reduces the polarity of the filter paper surface and consequently increases the interaction of the raw materials with the catalytically active surfaces in the modified filter paper. This, increases the contact time of the reactants with the filter paper during filtration and thus increases the efficiency.

The swelling amount of the filter papers was studied as an important parameter, because the high rate of swelling increases the contact time and interaction of the reactants with the filter paper (which contains catalytically active centers). The swelling rate for the prepared filter papers was given in Table 1.

**Table 1:** Swelling amounts of the plain FP, SiCFP, and FP@Si-Pd^II^-Salen-[IM]OH filter papers

Solvent		Swelling (g mL^−1^)								
		H_2_O	H_2_O : EtOH	DMF	EtOH	MeOH	DMSO	Toluene	Acetic acid	Acetone
Filter paper	Plain	8.8	11.2	13.3	6.8	7.5	14.2	0.6	5.9	1.5
	Silylated	10.0	15.2	14.0	9.1	9.6	15.7	0.6	7.2	3.0
	Filter paper 7	9.6	14.9	13.5	8.8	9.2	14.2	0.5	6.5	2.0

As shown in Table 1, the swelling amount in the silylated FP was significantly higher than in the plain filter paper, especially in protic solvents. The results were completely in agreement with the thermal behavior of the silylated filter paper with rigid and cross-linked structure that provides high swelling amount. The results of TGA also showed that the surface silylation, significantly increases the thermal resistance of the filter paper; But with the immobilization of the Pd^II^-salen complex, the swelling rate for all solvents decreased. As shown in Table 1, FP@Si-Pd^II^-Salen-[IM]OH showed the highest swelling amount in aqueous solvent: ethanol equal to 14.9 mL g^−1^.

### Catalytic activity

The catalytic activity of FP@Si-Pd^II^-Salen-[IM]OH filter paper in the Suzuki, Sonogashira and Heck coupling reactions was studied. Tables 2–4 show the results of these couplings in the presence of a wide range of aryl halides. Aryl iodides produced good to excellent conversion for all three coupling reactions. For aryl iodides, the number of filtrations at 0.3 bar gas pressure was between 3–5 times, which increased to 8 times, due to the low efficiency for aryl chlorides and aryl bromides. The results showed that the coupling reactions was not very desirable for aryl chlorides and requires more time to react. However, in order to increase the contact time of the reaction mixture with the catalytic filter paper, the gas pressure inside the Erlenmeyer for aryl chloride derivatives (for all three coupling reactions) was increased to 0.5 bar. Under these conditions, the coupling reactions were also performed for aryl chlorides. It should be noted that at the inlet gas pressure of 0.3 bar, due to the high number of filtration times for aryl chlorides and also the lack of optimal efficiency, the inlet gas pressure increased to 0.5 bar, which caused the reaction to progress. However, as shown in Tables 2–4, there was no efficiency for aryl chlorides bearing electron donor groups (such as Me and O–Me, Table 2, entries 2,3,7,8,14,15, Table 3, entries 2,3,9,10,17,18,22, Table 4, entries 2,3,9,10,16,17) even with inlet gas pressure of 0.5 bar and duration of 180 minutes (for 7 consecutive filtrations). For Suzuki coupling, % conversion was observed only with aryl chlorides bearing electron withdrawing groups (Table 4, entries 4,5,7,11,12,14,18,19).

**Table 2:** FP@Pd^II^-Salen-[IM]OH-catalyzed C–C Sonogashira cross-coupling reaction^a^

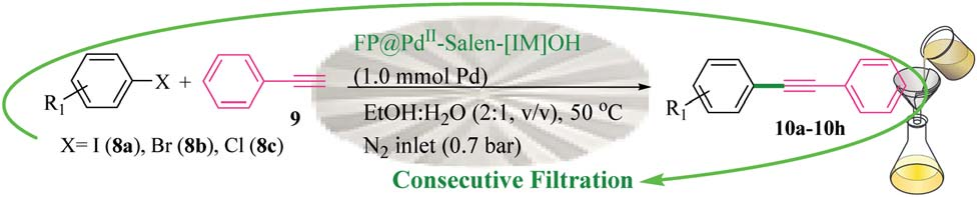				
Entry	Aryl halide	Product	Time (min)/Cycles^b^	Conversion^c^ (%)
1	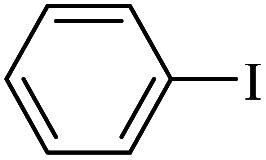	10a	70/5	96
2	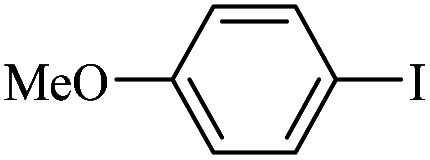	10b	107/7	86
3	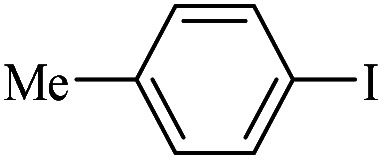	10c	107/7	88
4	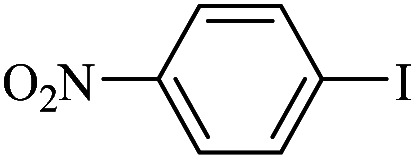	10d	70/5	96
5	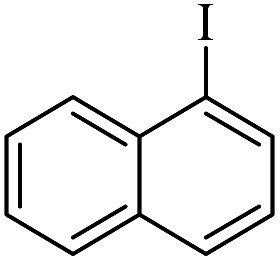	10e	107/7	90
6	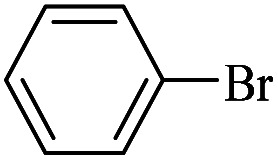	10a	110/4	80
7	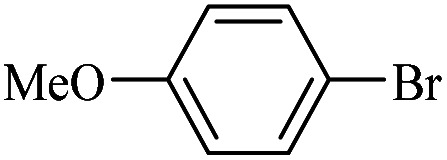	10b	143/5	75
8	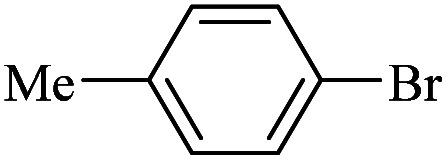	10c	143/5	75
9	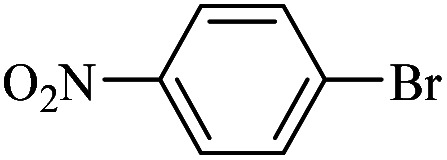	10d	80/3	85
10	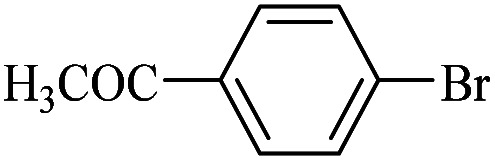	10f	80/3	85
11	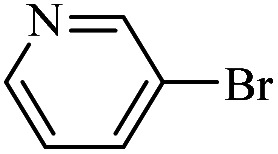	10g	80/3	86
12	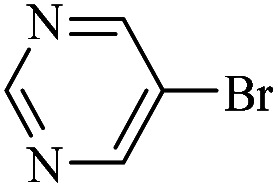	10h	80/3	88
13	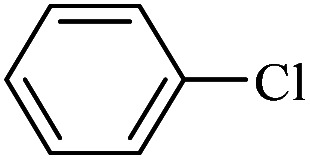	10a	180/7	50
14	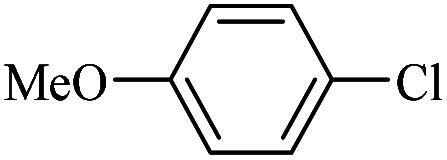	10b	180/7	N.R.
15	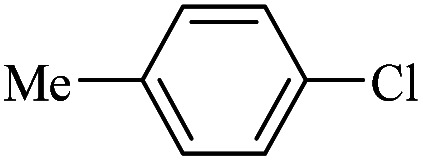	10c	180/7	45
16	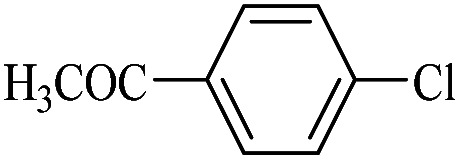	10f	180/7	60
17	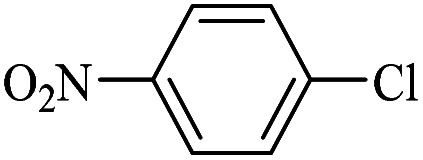	10d	180/7	65

a Reaction conditions: Phenylacetylene (1.0 mmol), iodobenzene (1.0 mmol), EtOH : H_2_O (2 : 1, v/v, 5.0 mL), 50 °C (on a water bath), N_2_ inlet (0.3 bar; 0.5 bar for aryl bromides, and chlorides), catalytic filter paper (7, placed on a glass funnel, containing 1.0 mmol Pd/95 cm^2^).

b Total time spent in different cycles. Cycles refers to number of re-filtration of the residue (Scheme 2).

c GC analysis.

**Table 3:** FP@Si-Pd^II^-Salen-[IM]OH-catalyzed C–C Heck cross-coupling reaction^a^

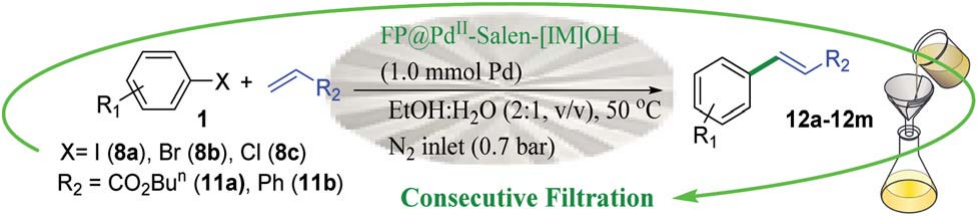					
Entry	Aryl halide	Alkene	Product	*t*. (min)/NOF^b^	Con^c^ (%)
1	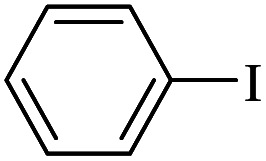	11a	12a	95/6	90
2	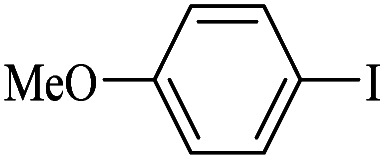	11a	12b	130/8	74
3	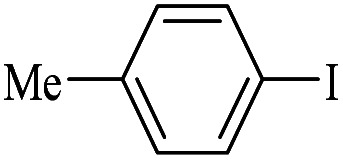	11a	12c	130/8	80
4	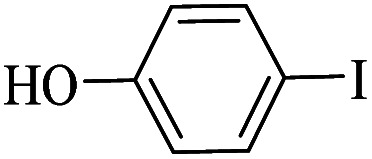	11a	12d	130/8	85
5	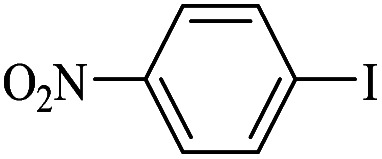	11a	12e	70/5	95
6	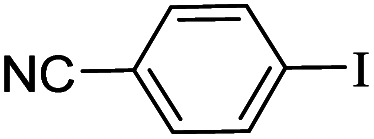	11a	12f	70/5	95
7	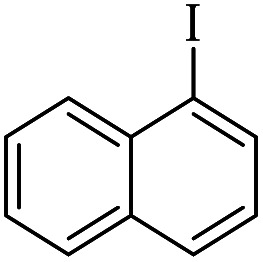	11a	12g	95/6	90
8	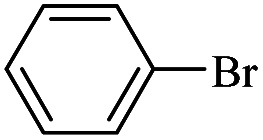	11a	12a	130/8	85
9	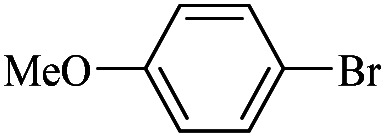	11a	12b	130/8	70
10	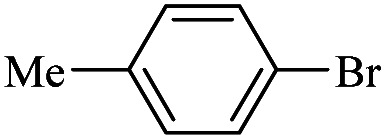	11a	12c	130/8	72
11	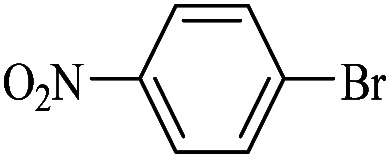	11a	12e	130/8	90
12	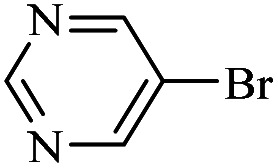	11a	12h	95/6	93
13	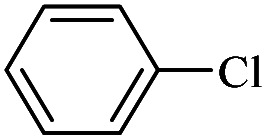	11a	12a	180/7	Trace
14	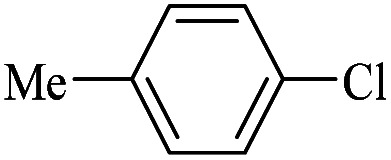	11a	12c	180/7	N.R.
15	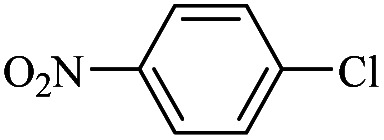	11a	12e	180/7	50
16	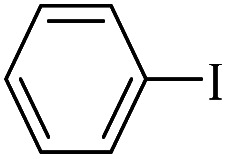	11b	12i	95/6	94
17	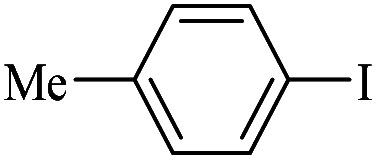	11b	12j	107/7	86
18	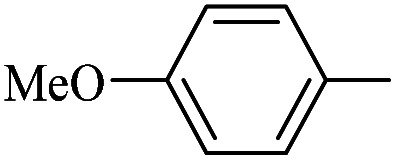	11b	12k	107/7	80
19	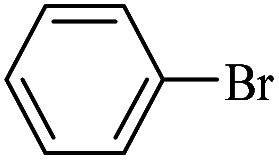	11b	12i	107/7	88
20	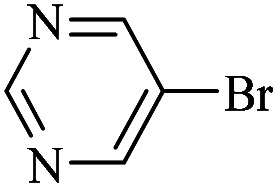	11b	12l	95/6	96
21	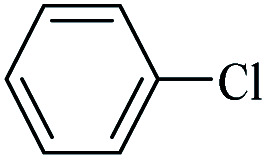	11b	12i	180/7	30
22	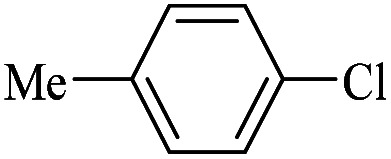	11b	12j	180/7	35
23	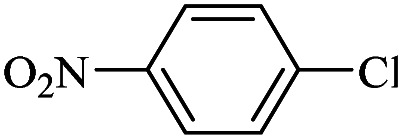	11b	12m	180/7	60

a Reaction conditions: Iodobenzene (1.0 mmol), styrene (1.0 mmol), EtOH : H_2_O (2 : 1, v/v, 5.0 mL), 50 °C (on a water bath), N_2_ inlet (0.3 bar; 0.5 bar for aryl chlorides), catalytic filter paper (7, placed on a glass funnel, containing 1.0 mmol Pd/95 cm^2^).

b Total time spent in different cycles. Cycles refers to number of re-filtration of the residue (Scheme 2).

c GC yield.

**Table 4:** FP@Si-Pd^II^-Salen-[IM]OH -catalyzed C–C Suzuki cross-coupling reaction^a^

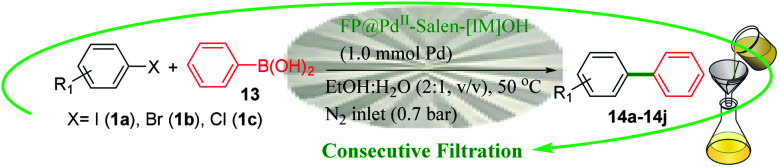				
Entry	Aryl halide	Product	Time (min)/No. of Filt.	Conversion^b^ (%)
1	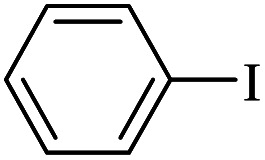	14a	70/5	95
2	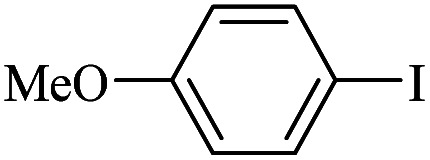	14b	70/5	80
3	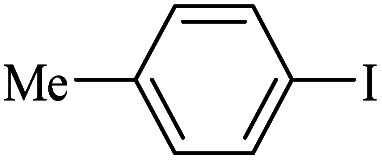	14c	70/5	80
4	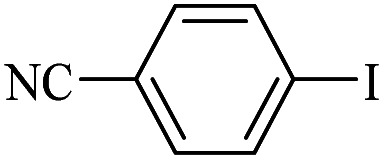	14d	54/4	96
5	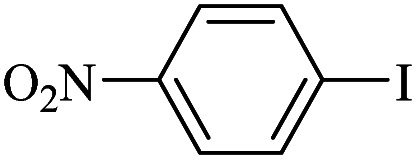	14e	54/4	96
6	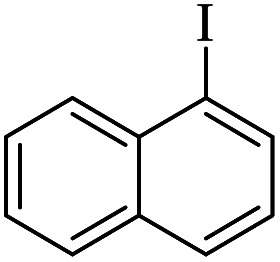	14f	70/5	92
7	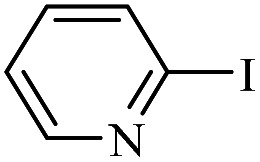	14h	54/4	95
8	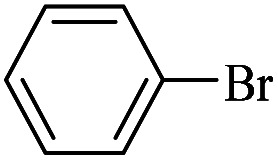	14a	107/7	75
9	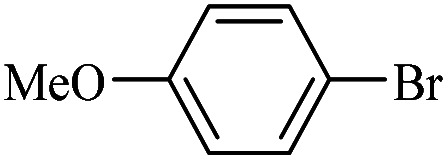	14b	130/8	70
10	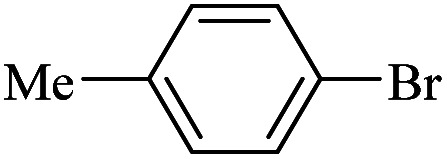	14c	107/7	70
11	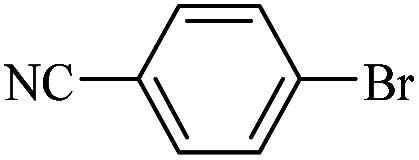	14d	107/7	85
12	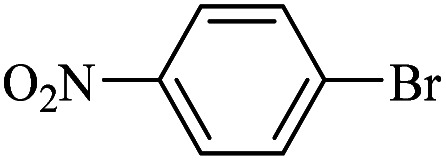	14e	107/7	85
13	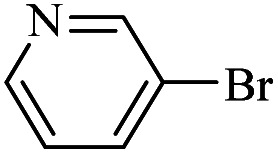	14i	107/7	86
14	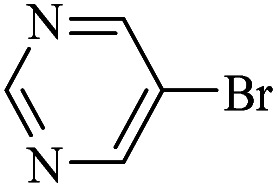	14j	107/7	82
15	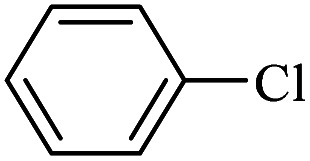	14a	180/7	N. R
16	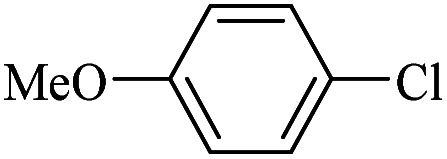	14b	180/7	N. R
17^c^	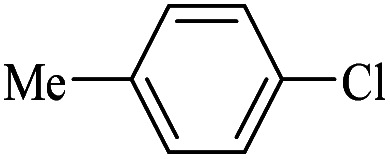	14c	180/7	N. R
18^c^	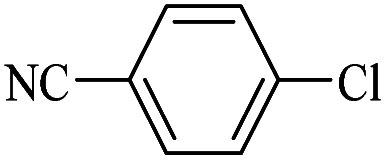	14d	180/7	45
19	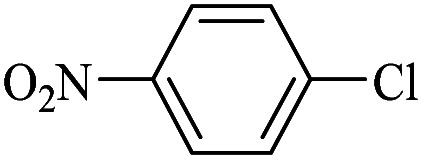	14e	180/7	40

a Overall reaction conditions: Phenylboronic acid (1.0 mmol), iodobenzene (1.0 mmol), EtOH : H_2_O (2 : 1, v/v, 5.0 mL), 50 °C (on a water bath), N_2_ inlet (0.3 bar; 0.5 bar for aryl chlorides), catalytic filter paper (7, placed on a glass funnel, containing 1.0 mmol Pd/95 cm^2^).

b Total time spent in different cycles. Cycles refers to number of re-filtration of the residue (Scheme 2).

c GC yield.

### Control experiments

To clarify the unique catalytic properties of FP@Si-Pd^II^-Salen-[IM]OH, the activity of various types of filter papers and different salts and compounds were studied over the Sonogashira model reaction. The first two experiments clearly elucidated the effectiveness of imidazolium moiety with OH counter ion (Table 5, entries 1,2). According to the results, the reaction was not only dependent on the presence of a basic reagent, but the reaction was catalyzed only by the presence of OH counter ions. It also appears that iodide does not have the ability to separate protons from their respective intermediates, or the formation of HI was not thermodynamically favorable relative to H_2_O (than the OH counter ion). The reaction efficiency in the presence of Pd^(II)^-salen-[IM]OH reached 60% (Table 5, entries 3), which has a significant difference with the activity of the catalytic filter paper (heterogeneous). This was a significant advantage over the Pd^(II)^-salen-[IM]OH homogeneous catalyst, which has the ability to catalyze the Sonogashira reaction in the green EtOH : H_2_O solvent by filtration. The presence of cellulose fibrous as well as imidazolium moiety-OH counter ion terminals in the filter paper causes proper diffusion of the reactants in this solvent between the cellulose fibrous containing the catalytic active sites and consequently provides a suitable environment for the coupling reaction (Refers to the proposed mechanism). PdCl_2_ salt also did not produce any detectable efficiency for the Sonogashira reaction (Table 5, entry 4). As expected, the filter paper alone did not produce any efficiency (Table 5, entry 5). Then, in order to prove the role of imidazolium-[OH] groups in the coupling reactions, a imidazolium-free filter paper was prepared in a similar way. The reaction under the exactly same conditions produced only 20% conversion. Despite the absence of any basic reagent (compared to Pd^II^-Salen complex 5, which did not produce any detectable efficiency), this amount of efficiency could be attributed to the appropriate environment created by the modified cellulose fibrous in the filter paper, which has the ability to give 20% conversion (Table 5, entry 6).

**Table 5:** Control experiments in the presence of some reagents as well as homologues of the filter paper 7 over the sonogashira cross-coupling model reaction^a^

Entry	Filter paper type/Pd complex	Time (min)	Conversion (%)
1	Pd^II^-salen complex^b^5	70	Trace
2	Pd^II^-salen-[IM]I	70	Trace
3	Pd^II^-salen-[IM]OH^b^	70	60
4	PdCl_2_ (anhydrous)^b^	70	N.R.
5	Plain filter paper^c^	70	N.R.
6	FP@Pd^II^-salen^c^	70	20
7	FP@salen-[IM]OH ligand^c^	70	N.R.

a Reaction conditions: Phenylacetylene (1.0 mmol), iodobenzene (1.0 mmol), EtOH : H_2_O (2 : 1, *v*/*v*, 5.0 mL), filter paper/Pd complex (as a catalyst).

b 1.0 mmol.

c For entries 1–3, the reactions were stirred using a magnet bar. For entries 4–6, the reactions were conducted on a water bath. All the reactions were conducted under N_2_ atmosphere and the Pd-loading was equal to 1.0 ± 0.3 mmol/95 cm^2^.

Preparation of a filter paper by immobilizing ligand 4 on the silylated filter paper (without the presence of any coordinated Pd) also did not produce any efficiency (Table 5, entries 7). The results of control experiments well showed that the components including Pd^II^-salen complex, the presence of imidazolium moieties along with the OH counter ions, immobilized on the cellulose fibrous were highly correlated with each other, so that the removal of each of them led to the loss of catalytic activity.

In the next step, the application of the catalytic FP was studied as small cut pieces or suspended in the reactions mixture for the preparation of 10a, 12a, and 14a. Table 6 shows the results of this study. As shown in Table 6, at similar time intervals, these two methods gave less efficiency than the filtration method for all three model reactions. Also, set-up 1 (using cut pieces of the filter paper in the reaction mixture) created higher efficiency than the suspended mode. The results showed well that the highest efficiency of the catalytic filter paper for the coupling reactions occurs in the filtration mode of the reactants and has a unique performance.

**Table 6:** Examination of two other set ups over the efficiency of Sonogashira (10a), Heck (12a), and Suzuki (14a) model reaction^a^

Set-up	12a		10a		14a	
	*t.* (min)	Con. (%)	*t.* (min)	Con. (%)	*t.* (min)	Con. (%)
Set up 1	95	84	70	80	70	80
Set up 2	95	50	70	55	70	65

a See Tables 2–4 for the reaction conditions. Set up 1 (Heterogeneous mode): 1 cm-square pieces of the cutted catalytic FP was used. Set up 2 (Suspended mode): Eight conical pieces of the cutted catalytic filter paper with a rim of 2.75 cm was suspended inside the reaction.

### Mechanism

By switching the N_2_ inlet gas to O_2_, the reaction efficiency of the Sonogashira model was drastically reduced. Due to the solubility of molecular oxygen in water, the catalytic filter paper dissolves the input molecular oxygen after absorbing water (along with ethanol as the reaction solvent). Molecular oxygen also binds to Pd active sites and prevents its activity. The control experiments showed that FP@salen-[IM]OH ligand had no catalytic activity and the Pd centers act as the catalytically active sites (Table 7, entry 6). Previously, Kazemnejadi *et al.* showed that the presence of imidazolium counter ions (OH or Cl) in the catalyst structure plays a base role and the coupling reaction proceeds in the absence of any external bases.^15–17,44^ The observations obtained from the control experiments in this study (Table 5), were also in complete agreement with the previous reports.^15–17,44^ As shown in Table 5 (entries 1,2), Pd^II^-salen complex 5 and Pd^II^-salen-[IM]I, did not give any efficiency for the Sonogashira model reaction; While 60% efficiency was found in the presence of Pd^II^-salen-[IM]OH for 10a.

**Table 7:** Reusability evaluation of the catalytic filter paper 7 using a sequential procedure consisting of three cross-coupling reactions of Sonogashira, Heck, and Suzuki towards the preparation of 10a, 12a, and 14a respectively using one filter paper^a^

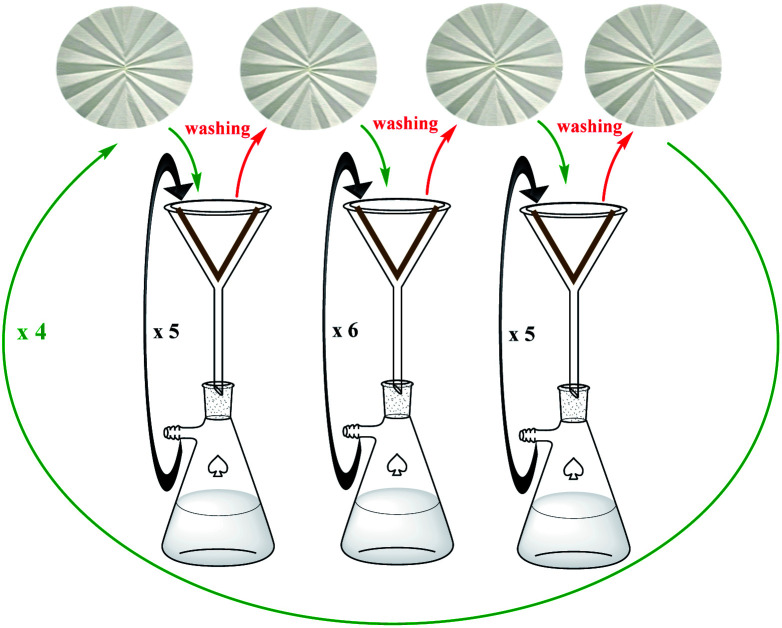						
Entry	Sonogashira reaction (10a)		Heck reaction (12a)		Suzuki reaction (14a)	
	*t* (min)/NOF.	Con. (%)	*t* (min)/NOF.	Con. (%)	*t* (min)/NOF.	Con. (%)
1st cycle	70/5	97	95/6	88	70/5	96
2nd cycle	70/5	95	95/6	86	70/5	94
3rd cycle	70/5	91	95/6	86	70/5	92
4th cycle	70/5	89	95/6	82	70/5	90

a In all experiments only one filter paper used.

**Scheme 3:**
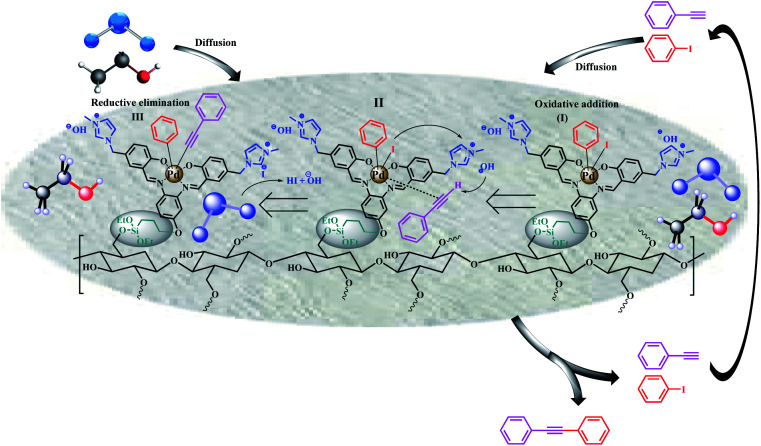
A plausible reaction mechanism for FP@Si-Pd^II^-Salen-[IM]OH catalyzed Sonogashira cross-coupling reaction (The mechanism could be extended for Heck and Suzuki cross-coupling reactions) Reductive elimination.

Scheme 3 shows the proposed mechanism in accordance with the literature and observations in this work. Reagents are first diffused into the filter paper by EtOH : H_2_O solvent. Due to the fact that very little concentration of the reactants enters the filter paper, the effective concentration inside the catalyst increases and causes a significant increase in efficiency. This phenomenon has already been observed for the catalysts based on PVA,^48^ sulfated zirconium oxide,^49,50^ and saponin^51^ with polar functional groups. Gravity causes this equilibrium to shift towards the filter paper. This phenomenon causes the reaction to proceed to completion and the effect of concentration does not affect the efficiency of the reaction too much.

After diffusion, the aryl halide (here iodobenzene) is attached to Pd *via* an oxidative-addition reaction (step I), that was in agreement with the previous reported mechanisms for the Pd-salen catalyzed coupling reactions.^52–54^ Phenylacetylene is coordinated to the Pd sites *via* a π bond, and by removing the proton by the hydroxyl group (counter ion of the imidazolium moiety), it binds to Pd and takes the substituent of iodide (step II). Iodide ion also acts as a counter ion of imidazolium moiety, which are then converted to HI and OH by water, and the catalyst returns to its original state. The control experiments also showed that FP@Pd^II^-Salen (as a homologue of the FP 7 without the imidazolium hydroxide moiety), does not produce significant efficiency for 10a, which confirms the function of this group as a basic agent (Table 5, entry 5). This step was confirmed by the recovery studies on the catalytic filter paper. As will be shown in the filter paper recovery studies in the next section, the wt% elemental composition (by EDX analysis) in the recycled FP@Si-Pd^II^-Salen-[IM]OH (after four consecutive recoveries) was quite similar to the freshly prepared FP@Si-Pd^II^-Salen-[IM]OH, and no trace of iodide was observed. In addition, the decrease in the catalytic activity of FP@Si-Pd^II^-Salen-[IM]OH during several consecutive recoveries was negligible.

In the third step, by reductive-elimination,^52–54^ the coupling product passes through the filter paper due to its solubility in ethanol (Step III). On the other hand, due to the higher efficiency of aryl halides bearing electron withdrawing group compared to the halides with electron donating substituent (Tables 2–4), it was suggested that the reaction proceed from the path including oxidative-addition and reductive-elimination steps in agreement with the literature.^18,47,55^

As shown in Tables 2–4, the efficiency as well as the filtration time and cycles for aryl halides were as order of I > Br > Cl, and even for some aryl chlorides no efficiency was observed (Table 2, entry 14; Table 3, entries 13,14; and Table 4, entries 15–17). These results were a strong evidence for the oxidative-addition and reductive-elimination steps.^18,47,52–54^

### Recyclability and stability of the filter paper

A significant advantage of the modified filter paper bearing Pd^II^-salen complex was its stability and reusability in the C–C coupling reactions. The catalytic activity of FP@Si-Pd^II^-Salen-[IM]OH in different cycles in the models coupling reactions of (1) coupling of iodobenzene with phenylacetylene, (2) coupling of iodobenzene with phenylboronic acid, and (3) coupling of iodobenzene with styrene, was studied. Catalyst recoverability was studied using one filter paper for all studies. Table 7 shows how these studies were performed. Using one filter paper, 10a, 12a, and 14a coupling products were prepared for four consecutive cycles. After each reaction, the filter paper was washed with water and ethanol and used immediately for the next reaction. As shown in Table 7, no significant drop in catalytic activity was observed for the reactions up to the fourth cycle, and the filter paper appears to be reliable for several more cycles. The significant advantage of FP@Si-Pd^II^-Salen- [IM]OH over heterogeneous catalysts was that there was no mass loss of catalyst, which means that no significant drop in the FP performance was observed during successive cycles.

In addition, as will be discussed below, the filter paper owes this high stability due to the presence of silica groups as well as Pd^II^-salen complex on the FP surface, which minimizes the shrinkage, and consequently preserves swelling due to the high stability of the filter paper.

Another advantage was the lack of leaching of any metal (especially Pd) as a result of residual solution analysis. In each reaction (after completion of the reaction), the remaining solution was investigated by ICP-MS to determine the leaching of Si and Pd. The analyzes did not show any trace of Si and Pd metals until the end of the fourth cycle, which reflects the absence of any metal leaching in the solution. In fact, the catalyst was designed in such a way that it does not create any soluble part due to the contact and successive passage (filtration) of solvents, and this issue, along with the stable coordination of Pd to the ligand framework, has caused no metal leaching from the FP. Therefore, the slight decrease in efficiency can be related to the decrease in FP quality in successive wetting–drying cycles. As will be shown below, during successive wetting–drying cycles, the plain paper undergoes some shrinkage and reduced swellability.

**Scheme 4:**
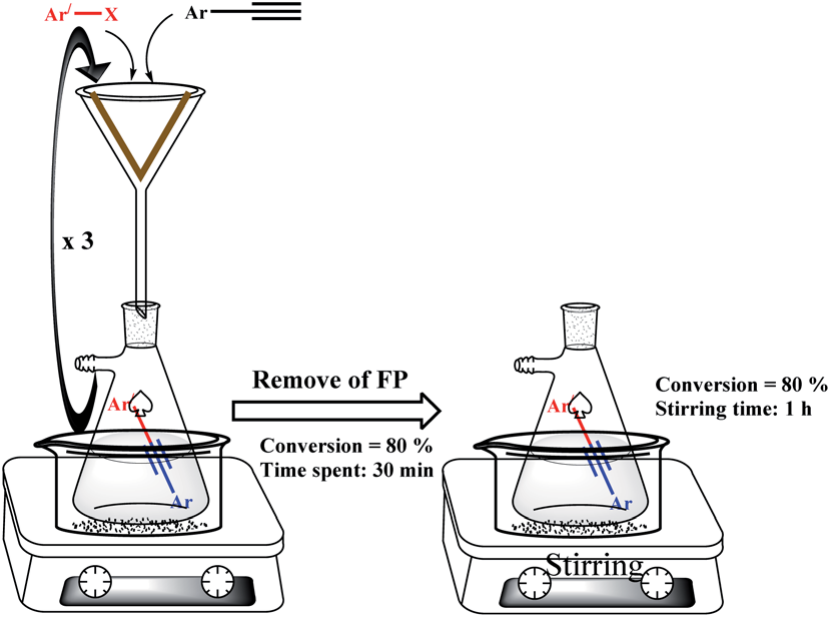
Metal leaching determination of FP 7 using the hot filtration assay.

In addition, the hot filtration test was also studied on the catalytic filter paper. For this purpose, in the model Sonogashira reaction, after the third filtration (80% conversion, 30 minutes), the filtration was stopped and the filter paper was removed (Scheme 4). Then, a magnet was added to the collected mixture and stirred at 50 °C. GC analysis of the reaction mixture after 1 hour also recorded an efficiency of 80%. The results showed well that despite successive filtrations, no Pd leaching occurs due to the coordination to a stable salen ligand structure. The results of EDX and leaching analyses were also completely consistent with these results and confirm the stability, no contamination, and reproducibility of the modified filter paper.

**Fig. 6:**
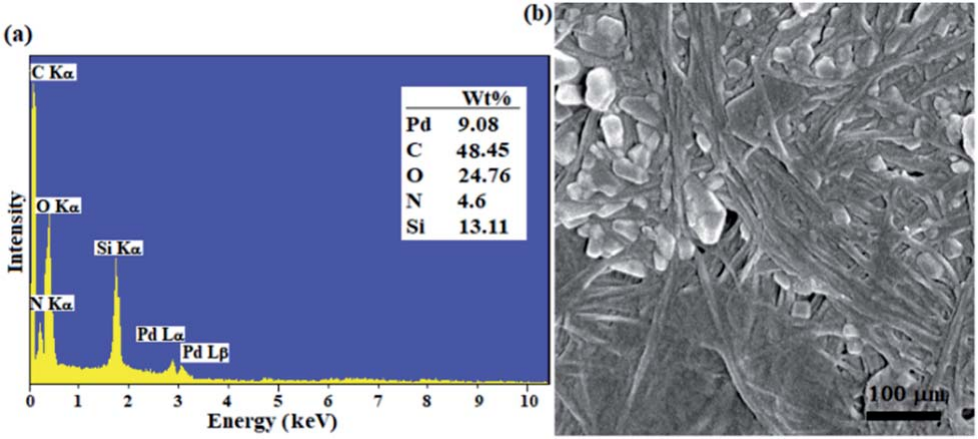
(a) EDX analysis (inset Table represents the elemental composition results in mean of 5 points) and (b) FESEM image of FP@Si-Pd^II^-Salen-[IM]OH after 4 consecutive recycles.

In addition, the FP was characterized by FESEM and EDX analyses after the 4th cycle in the reaction of Sonogashira model (coupling of phenylacetylene with iodobenzene). This analysis was performed to ensure the adsorption of any impurities (including raw materials and products) on the filter paper after repeated use. As shown in Fig. 6a, the % wt of C, O, N, Pd elements has not changed significantly, which reflects the filtration and subsequent complete washing of the filter paper after filtration. In addition, this analysis showed that the absence of any impurities in the filter paper after the reaction allows its repeated use with confidence. In addition, the FESEM image of the recovered filter paper (Fig. 6b) showed that its morphology did not change compared to the freshly prepared filter paper. The results demonstrating the high stability of the immobilized palladium complex on the FP through strong covalent bonding.

To investigate the stability of the immobilized groups on the filter paper, various reagents were studied for this purpose, passed through the filter paper and the filtrate as well as the filter paper were analyzed. Table 8 shows the effect of acidic, alkaline and oxidative reagents on the elemental composition of the filter paper. Also, to investigate the possible leaching of Si and Pd metals on the filter paper, the filtrate was examined using ICP-MS.

**Table 8:** Stability study of the FP@Si-Pd^II^-Salen-[IM]OH against acidic, basic, and oxidative reagents^a^

Entry	Sample	Conditions	ICP or EDX result (%wt)				
			C	N	Pd	Si	O
1	Residue (ICP analysis)	NaOCl	—	—	0	0	—
2		HNO_3_ (0.1 N)	—	—	4.88	6.97	—
3		HCl (0.1 N)	—	—	1.33	0.19	—
4		NaOH (0.1 N)	—	—	0	0	—
5		H_2_O_2_ 37%	—	—	0	0	—
6	FP@Si-Pd^II^-Salen-[IM]OH (EDX analysis)	NaOCl	50.20	4.77	5.16	12.99	26.88
7		HNO_3_ (0.1 N)	55.12	2.21	0.26	6.88	35.53
8		HCl (0.1 N)	53.19	4.56	5.06	12.50	24.69
9		NaOH (0.1 N)	50.23	4.80	5.23	12.81	26.93
10		H_2_O_2_ 37%	50.17	4.83	5.22	12.79	26.99

a 5 mL of each reagent was filtered for ten consecutive times at ambient temperature.

The filter paper stability studies provided useful information on behavior of the filter paper in various media. Table 8 shows the results of this study. Nitric acid causes significant leaching of Pd into the solution. In addition, due to the reduction in the percentage of nitrogen in the resulting filter paper, it appears that the Schiff base ligand was also hydrolyzed in an acidic medium and enters the solution phase (Table 8, entries 2 and 7). The filter paper has complete stability in basic environment (NaOH 0.1 N) which shows its applicability in high pH solutions. No traces of Si and Pd in the remaining solution were detected by ICP-MS (Table 8, entries 4 and 9). In the presence of HCl 0.1 N, the filter paper has a relative stability, so that the leaching rate was negligible for both Pd and Si metals (Table 8, entries 3 and 8). Lack of effect of oxidants such as NaOCl and H_2_O_2_ on the composition of the filter paper reflects the high stability of the filter paper structure against oxidants and therefore makes it possible for use in oxidation and epoxidation reactions (Table 8, entries 1, 5, 6, and 10).

In order to investigate the possibility of using the FP at different pHs, the amount of Pd leaching from the filter paper at pHs 1–14 (using HCl and NaOH solutions) was studied. Fig. S14† shows the amount of Pd leaching at each pH after 5 consecutive filtrations of each solution by ICP analysis. As shown in Fig. S14,† the highest Pd leaching rate occurs at pH 1 equal to 4.89%. At this pH, almost all the Pd loaded on the filter paper was leached. The filter paper was relatively stable at pHs 5–11 and no metal leaching was detected and the catalytic filter paper can be used with confidence in this pH range.

Schiff base bond hydrolysis at very acidic and highly alkaline pHs was the main cause of the high leaching rate observed at these pHs. However, this stability was much higher in the acidic environment than in the basic, so that at pH 14 only 4.2 % Wt Pd leaching was detected.

**Fig. 7:**
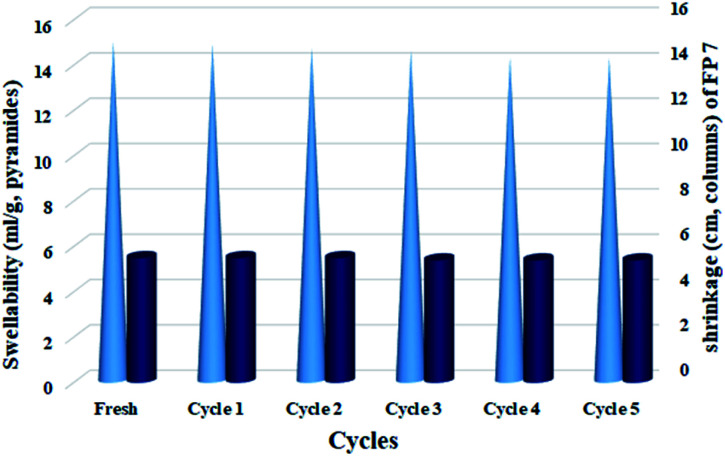
Swellability and shrinkage measurements of FP@Si-Pd^II^-Salen-[IM]OH in EtOH : H_2_O (2 : 1, *v*/*v*) for 5 consecutive wetting–drying cycles.

In order to study the effect of successive drying-wetting of the filter paper on the shrinkage and its swelling rate, the catalytic filter paper was washed and dried for 5 consecutive times by EtOH : H_2_O (2 : 1) solution and its shrinkage and swelling rate in each cycle was measured. The results show that the physical properties of the cellulosic fibers were affected by successive drying-wettings,^56^ and these properties can be changed by proper functionalization of the fibers.^57,58^Fig. 7 shows the results of this study. According to the results, the amount of swelling in the catalytic filter paper did not change significantly during successive drying-wetting cycles, which reflects the high stability of the filter paper arising from the proper modification with organic groups, especially silica supported groups.

The results were completely in agreement with the thermal behavior of the silylated filter paper (Fig. 2b). Also, the amount of shrinkage remained constant until the end of the 5th drying-wetting cycle and there was no significant reduction in the paper diameter (Fig. 7).

The amount of shrinkage observed in each run was negligible and at the end of the 5th run, only 1 mm decrease in the size of the filter paper diameter was observed. This stability in the filter paper can be directly attributed to the presence of silica groups as well as the Pd-salen complex immobilized on the filter paper. The study of shrinkage and swelling amounts on the plain and the silylated filter paper confirmed the effect of these two factors on maintaining the properties of filter paper.^19^ According to the results, the plain filter paper suffers from 7 mm shrinkage, and the swelling rate was reduced to 8.6 mL g; While these values were equal to 2 mm and 12 mL g^−1^ respectively, for the silylated filter paper. The results showed a correlation between these two parameters that with the suitable surface functionalization, both improve.^19^

### Comparison of activity of FP 7 with literature reports

Table S9† shows the performance and catalytic properties of FP@Si-Pd^II^-Salen-[IM]OH filter paper compared to the previously reported coordinated Pd-based heterogeneous catalysts for the preparation of 10a (coupling reaction of phenylacetylene with iodobenzene).^59–64^ As shown in Table S9,† FP@Si-Pd^II^-Salen-[IM]OH was superior to most previously reported heterogeneous catalysts in terms of ease of work-up, environmentally friendly, and the product efficiency. FP@Si-Pd^II^-Salen-[IM]OH was prepared on a cheap and plain cellulose filter paper using the available raw materials and was portable. Unlike nanomaterials-based heterogeneous catalysts, FP@Si-Pd^II^-Salen-[IM]OH filter paper can be recovered without metal leaching for several consecutive cycles. Heterogeneous catalysts based on nanomaterials and transition metal complexes often have metal leaching that allows toxic metals such as Pd to enter the environment. In addition, the recovery of FP@Si-Pd^II^-Salen-[IM]OH was done with just a simple washing and, therefore, no mass was lost from the catalyst, while the recovery of nanomaterials-based heterogeneous catalysts is often done by centrifugation which the loss of catalyst during successive recovery cycles due to their nano-dimensions is inevitable and causes a decrease in catalytic properties. As shown in Table S9,† the recovery of heterogeneous catalysts is usually done using methods such as a magnet, filtration, extraction, and centrifuge, which either cause the catalyst to be lost or contaminated.^59–64^ In addition, contamination of heterogeneous catalysts with raw materials and products in each cycle requires tedious and continuous washing.^59–64^ These disadvantages also limit the use of nanomaterial-based heterogeneous catalysts for use at higher scales. The FP@Si-Pd^II^-Salen-[IM]OH potable catalyst can be used anywhere, even by non-experts. FP@Si-Pd^II^-Salen-[IM]OH has a multiple function, which owes it to the immobilization of the Pd-salen complex, so that the Sonogashira reaction takes place in the absence of Cu salt (as a co-catalyst), a toxic and expensive phosphine ligand, and a base agent. These factors make the filter paper more environmentally friendly. FP@Si-Pd^II^-Salen-[IM]OH as a portable catalyst, eliminates most of the disadvantages associated with heterogeneous catalysts, not only providing better efficiency than heterogeneous catalysts, but also easy recovery and no metal leaching, sustaining the environment.

## Conclusion

A Pd^II^-salen complex-functionalized cellulose FP was used as an efficient portable catalytic filter paper for the construction of C–C bonds *via* the Heck, Suzuki and Sonogashira reactions based on the filtration and controlled passage of the reactants. The modified FP was characterized by ATR, EDX, XPS, TGA, XRD, and FESEM analyses. High to excellent yields were obtained for the coupling reactions with an average of 3–8 filtration cycles for 54–180 min. The Pd^II^-salen complex was covalently bonded to a pre-silylated filter paper, which were responsible for high stability and revocability by maintaining the catalytic activity with no leaching and shrinkage during successive wetting–drying cycles. The filter paper was stable in a wide range of acidic pHs (5, 6) and alkalis (8–11) and thus can be used in a wide range of organic reactions. Sustainability, portable, easy work-up, clean profile, reusability, high stability, high versatility, high catalytic activity and selectivity (as much as known heterogeneous catalytic systems), *etc.* were some of the present novel protocol, which make it as a promising candidate and alternative than traditional heterogeneous catalytic systems. As best of our knowledge, this is the first report of coupling reactions catalyzed by filtration, and further studies based on various modifications on the filter paper could create a new world for portable catalysts.

## Experimental

### Materials and methods

A circle Whatman 1201–320, grade 1V cellulose filter paper (folded), with a 32 cm in diameter, 150 s/100 mL speed (Herzberg) 0.2 mm thickness, 8–10 micron, with basic weight of 120 g m^−2^ was provided from Sigma. To control of filtration rate (the duration of contact time), the reaction set up was equipped by a N_2_-inlet (low pressure: 0–1 bar) V-121K091 R-21 regulator (inlet connection W24,32). Orbital Shaker, 10 mm Orbit from BTLabSystems was used for shaking. ATR (in the case of filter papers) and FTIR analyses were taken on a JASCO FT/IR 4600 instrument. The NMR (^1^H and ^13^C) spectra were recorded on a Bruker AVANCE III 300 MHz spectrometer in deuterated solvents (CDCl_3_ and DMSO-*d*_6_). NexION 2000B ICP Mass Spectrometer was used for ICP analyses. X-ray diffraction (XRD) of the FPs was studied on a Rigaku Smart-Lab X-ray diffractometer with Cu *K*α (*λ* = 1.5418 nm) radiation. Field emission scanning electron microscopy (FESEM) of the FPs were taken using a Tescan MIRA3 microscope. Energy-dispersive X-ray spectroscopy (EDX) was conducted on a JEOL 7600F field emission scanning electron microscope, equipped with a spectrometer of energy dispersion of X-ray from Oxford Instruments. Statistical studies on the coupling parameters and also subsequent regression analyses of the experimental data were studied by a Design-Expert statistical software version 11, Stat-Ease Inc. Minneapolis, MN, USA.

#### Synthesis of 2-hydroxy-5-(iodomethyl)benzaldehyde (SA-5-IM, 2)

In first, 2-hydroxy-5-chloromehyl benzaldehyde (SA-5-ClM) was prepared according to the previously reported procedures (purple crystals, 97% isolated yield).^15–18^ Then, in order to synthesis of 2-hydroxy-5-(iodomethyl)benzaldehyde (named as SA-5-IM briefly), SA-5-ClM (2.0 mmol) was dissolved in dry acetone (20 mL), then 4.0 mmol of Na i was added to the mixture. The mixture was stirred at ambient temperature for 2.0 h. Then, the mixture was filtrated by a simple Whatman No.1 filter paper to remove of NaCl salts, then SA-5-IM product was obtained by removing the solvent at room temperature (88% isolated yield).

The change of the halide substituent from Cl to I was performed based on a part of the Hoffman elimination and the formation of the hydroxide counter ions on the ionic moiety.^17,44^ Experiments have shown that the formation of chloride groups (imidazolium chloride) in the presence of Ag_2_O does not produce any efficiency for the imidazolium chloride and its subsequent conversion to the hydroxide counter ions, but in the presence of iodine counter ions, the reaction proceeds well, in complete agreement with the previous published articles.^17,44^

Characterization data for SA-5-IM (2): Pale yellow powder; M.P. 114 °C; EDX analysis (%wt): C, 36.71; I: 48.34, O: 12.23; Anal. Calcd. for C_8_H_7_IO_2_ : C, 36.67; H, 2.69%; Found: C, 36.64; H, 2.73%.

#### 2,2′-(1*E*,1′*E*)-((4-hydroxy-1,2-phenylene)bis(azanylylidene))bis(methanylylidene))bis(4-(iodomethyl)phenol) [*N*,*N*′-(4-OH-phenylenebis(SA-5-IM), 3]

*N*,*N*′-(4-OH-phenylenebis (SA-5-IM)) 3 was simply prepared with dissolution of SA-5-IM (2.0 mmol) and 3,4-diaminophenol (1.0 mmol) in 20 mL dry EtOH under Ar atmosphere. The reaction was refluxed for 4 h with constant stirring. The resulting product was obtained after filtration of the cooled solution. The product was washed with cooled EtOH and deionized water, then dried at 55 °C in an vacuum oven and isolated as a yellow solid.

Characterization data for *N*,*N*′-(4-OH-phenylenebis(SA-5-IM)), 3: Yellow solid; M.P. 174 °C; EDX analysis (%wt): C, 43.22; I: 41.33, O: 7.88, N, 4.58; Anal. Calcd. for C_8_H_7_IO_2_ : C, 43.16; H, 2.96%; N, 4.58%; Found: C, 43.06; H, 3.00%, N, 5.04.

#### Synthesis of [*N*,*N*′-(4-OH-phenylenebis(SA-5-ImI)), 4]

For the preparation of ionic compound 4 as a ligand, [*N*,*N*′-(4-OH-phenylenebis(SA-5-ImI))], *N*,*N*′-(4-OH-phenylenebis(SA-5-IM)) (2.0 mmol) and 4.0 mmol of 1-methylimidazole were dissolved in dry ethanol. The reaction was refluxed under Ar atmosphere for 3.0 h. Then, the resulting light orange solid was filtered after cooling the reaction mixture, and then washed with deionized water as well as 1.0 mL of cool EtOH.

Note: Different synthetic routes were evaluated for the synthesis of 5, and the routes shown in Scheme 1 was chosen as the most promising and repeatable with the highest efficiency for the preparation of catalytic filter paper 7.

#### Synthesis of [*N*,*N*′-(4-OH-phenylenebis(SA-5-ImI))-Pd^(II)^complex, 5]

Complexation of Pd ion to *N*,*N*′-(4-OH-phenylenebis(SA-5-ImI)) ionic ligand was performed simply by dissolution of 2.0 mmol of ligand 4 and 1.0 mmol of PdCl_2_ to ETOH (20.0 mL) at ambient temperature. The reaction was stirred for 2.0 h. The resulting brown solid was filtered, washed with deionized water as well as cooled EtOH, then isolated as a stable light brown powder at room temperature for the next step.

#### Silylation of cellulose filter paper

Cellulose FP silylation was performed using a solution method (in water) that was described in our previous work in detail.^19,28^

#### Preparation of 4-OH-phenylenebis(SA-5-ImI)-Pd^(II)^complex-embedded filter paper (FP@Si-Pd^II^-Salophen)

Initially, 0.2 mmol Ag_2_O was added to an ethanolic ammonia solution (15 mL) in a 6 cm diameter glassy petri dish. The role of Ag_2_O was in the converting iodide ions to hydroxides as a part of the Hoffman elimination.^44^ Next, 0.1 mmol 4-OH-phenylenebis(SA-5-ImI)-Pd^(II)^ complex (200 mg) was added to the mixture in a petri dish, and the SiCFP was slowly inserted into the solution in the petri dish. To covalent functionalization of the filter paper with the palladium complex, the solution was shacked at ambient temperature for 2 days. The FP@Si-Pd^II^-Salophen was dried and stored in a vacuum desiccator containing P_2_O_5_.

#### Typical procedure for FP@Si-Pd^II^-Salen-[IM]OH catalyzed C-C cross-coupling reactions

As a typical procedure for the FP@Si-Pd^II^-Salen-[IM]OH catalyzed C–C Sonogashira cross-coupling reaction, phenylacetylene (1.0 mmol) and iodobenzene (1.2 mmol) was dissolved in a solution of EtOH : H_2_O (2 : 1, *v*/*v*, 5 mL) at ambient temperature. FP@Pd^II^-Salen was placed on a glass funnel, that was set-up on a vacuum Erlenmeyer flask equipped with a N_2_ inlet (0.3 bar) (Scheme 2). The reaction set up was placed on a water bath adjusted at 50 °C. The reaction was performed by filtration of the reactants. In each filtration run, the reaction mixture was poured on the filter paper in one step, and the filtration time was calculated from the moment of pouring the reaction mixture on the funnel to the filtration of the last drop, and the total time was considered as the reaction time. The reaction progress was monitored by GC and/or TLC from the filtrate. Finally, the used FP was washed with hot absolute ethanol (10 mL) and the residue was added to the mixture to calculate the reaction efficiency.

#### Swelling measurements of filter papers

The swelling amounts of the FPs were determined by weight changes in the film in the solvents^65,66^ using eqn (1):1
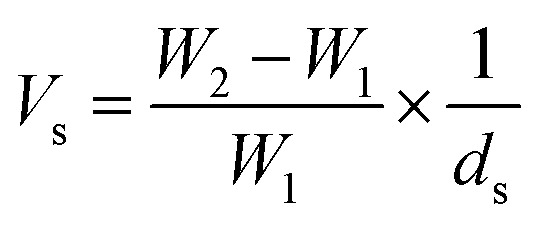
where *V*_s_, *W*_2_, *W*_1_ and *d*_s_ are swelling amount of the filter paper (mL g^−1^), weight of swollen network (gr), weight of dry filter paper (gr) and density of the solvent, respectively.

#### Other procedures

Two other protocols were also examined for set-up of the reaction to evaluate the catalytic activity of the FP *via* the filtration method including, of (1) suspending the filter paper inside the reaction mixture;^2^ and (2) use of the cutted FP species in the reaction mixture (like a heterogeneous catalyst).

(1) In the first set up, the FP was suspended inside the reaction mixture using a clamp according to a previously described procedure by Nishikata *et al.*^2^ Given that the Pd loading was depends on the surface area of the FP, one complete sheet of the FP (with diameter of 5.5 cm) was used for this test to make a logical comparison with the other methods. For this purpose, a sheet of the FP was divided into 8 equal cone-shaped parts with a 2.75 cm in rim and then, suspended into the mixture, and the mixture was stirred magnetically and the reaction progress was monitored by TLC or GC continuously. After the reaction completion, the recovered FP pieces were shaken in absolute EtOH (15 mL) to achieve the highest efficiency, and the resulting solution was added to the final mixture to calculate the reaction efficiency.^19^

(2) In the second set up, the FP was cut into several square pieces with 1 cm in diameters and added to the reaction mixture. The mixture was stirred by a magnet and its progress was monitored by TLC or GC analysis. Upon the reaction completion, the FP species were separated from the reaction mixture by forceps and shaken for 5 h along with a 15 mL of EtOH, and the resulting ethanolic mixture was added to the main mixture to calculate the reaction conversion.

## Author contributions

All authors contributed to the study conception and design. Material preparation, data collection and analysis were performed by Indah Raya, Svetlana Danshina, Wanich Suksatan, Mustafa Z. Mahmoud, Ali B. Roomi, Yasser Fakri Mustafa. The first draft of the manuscript was written by Milad Kazemnejadi and Abduladheem Turki Jalil and all authors commented on previous versions of the manuscript. All authors read and approved the final manuscript.

## Conflicts of interest

There are no conflicts to declare.

## Supplementary Material

RA-012-D2RA03440A-s001
