# Supplementary material for: Catalytic filtration: efficient C-C cross-coupling using Pd(II)-salen complex-embedded cellulose filter paper as a portable catalyst
Source: RSC Adv. 2022 Jul 12;12(31):20156–73. doi: 10.1039/d2ra03440a (PMC9274805; doi:10.1039/d2ra03440a)
Supplement: RA-012-D2RA03440A-s001 [file RA-012-D2RA03440A-s001.pdf]

Electronic Supplementary Material (ESI) for RSC Advances.
This journal is © The Royal Society of Chemistry 2022

Supporting Information

Catalytic filtration: Efficient C-C cross-coupling using a Pd$^{(II)}$-salen complex-embedded cellulose filter paper as a portable catalyst

Indah Raya,[a] Svetlana Danshina,[b] Abduladheem Turki Jalil,[c,d,e]* Wanich Suksatan,[g] Mustafa Z. Mahmoud,[h,i] Ali B. Roomi,[j,k] Yasser Fakri Mustafa,[l] Milad kazemnejadi*[m]

[a] *Departement of Chemistry,Faculty Mathematics and Natural Science, Hasanuddin University, Makassar,South Sulawesi,90245 Indonesia.*
[b] *Sechenov First Moscow State Medical University, Moscow, Russia.*
[c] *Faculty of Biology and Ecology, Yanka Kupala State University of Grodno, 230023 Grodno, Belarus.*
[d] *College of technical engineering, The Islamic University, Najaf, Iraq.*
[e] *Department of Dentistry, Kut University College, Kut, Wasit, 52001, Iraq.*
[f] *Faculty of Nursing, HRH Princess Chulabhorn College of Medical Science, Chulabhorn Royal Academy, Bangkok, Thailand.*
[g] *Department of Radiology and Medical Imaging, College of Applied Medical Sciences, Prince Sattam bin Abdulaziz University, Al-Kharj 11942, Saudi Arabia*
[h] *Faculty of Health, University of Canberra, Canberra, ACT, Australia.*
[i] *Ministry of Education, Directorate of education Thi-Qar, Thi-Qar, 64001, Iraq.*
[j] *Biochemistry and biological engineering research group, Scientific Research Center, Al-Ayen University, Thi-Qar, 64001, Iraq.*
[k] *Department of Pharmaceutical Chemistry, College of Pharmacy, University of Mosul, Mosul, Iraq.*
[l] *Department of Chemistry, Faculty of Science, Golestan University, Gorgan, Iran.*
Corresponding author: abedalazeem799@gmail.com (A. T. Jalil); miladkazemnejad@yahoo.com (M. Kazemnejadi)

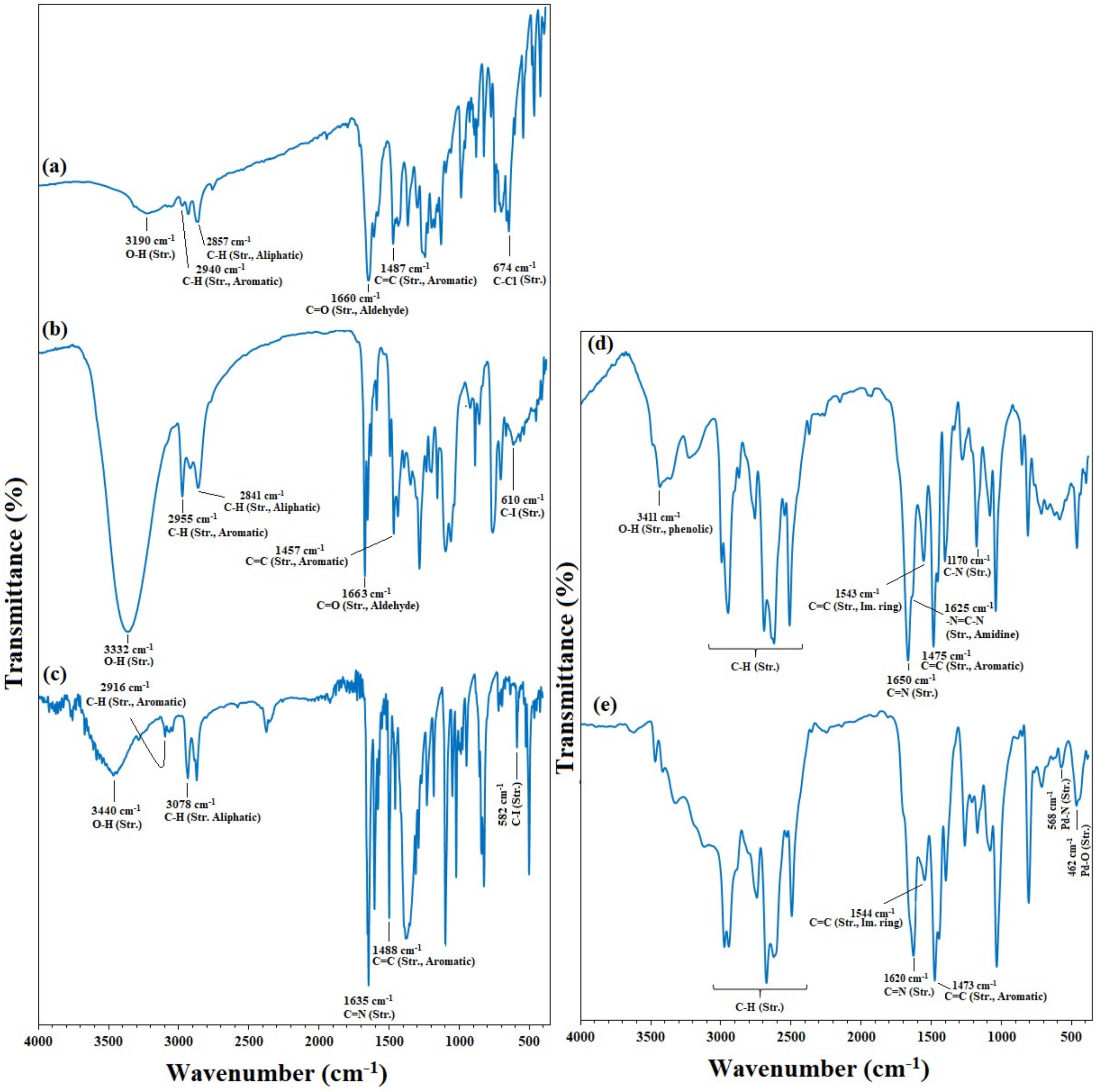

Fig. S1 FTIR spectra of (a) 5-chloromethyl salicylaldehyde (**1**), (b) 5-iodomethyl salicylaldehyde (**2**), (c) salen ligand (**3**), (d) salen-[IM]I ligand (**4**), and (e) Pd(II)-salen-[IM]I complex (**5**)

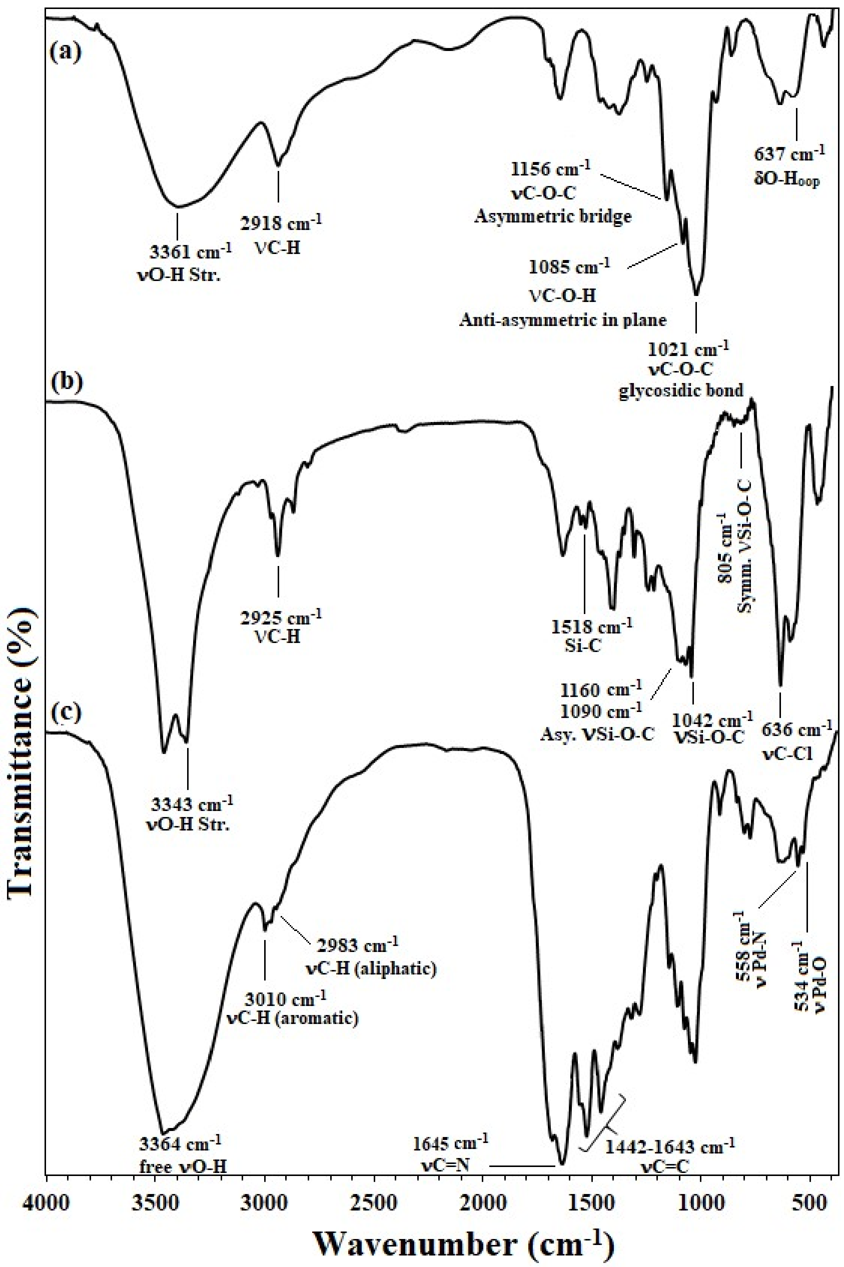

Fig. S2 ATR-IR spectra of (a) plain (unmodified) cellulose filter paper, (b) SiCFP, and (c) FP@Si-Pd^{II}-Salen-[IM]OH

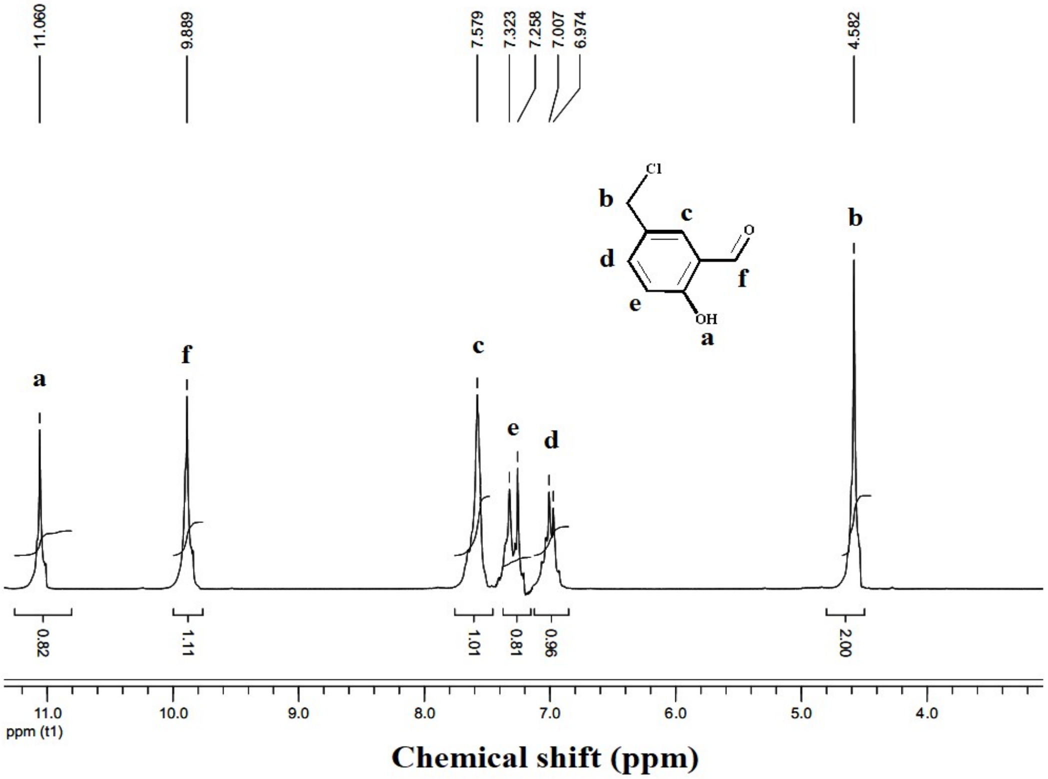

Fig. S3 ^{1}H-NMR spectrum of 5-chloromethyl salicylaldehyde (**1**) ($CDCl_3$, 300 MHz) [1]

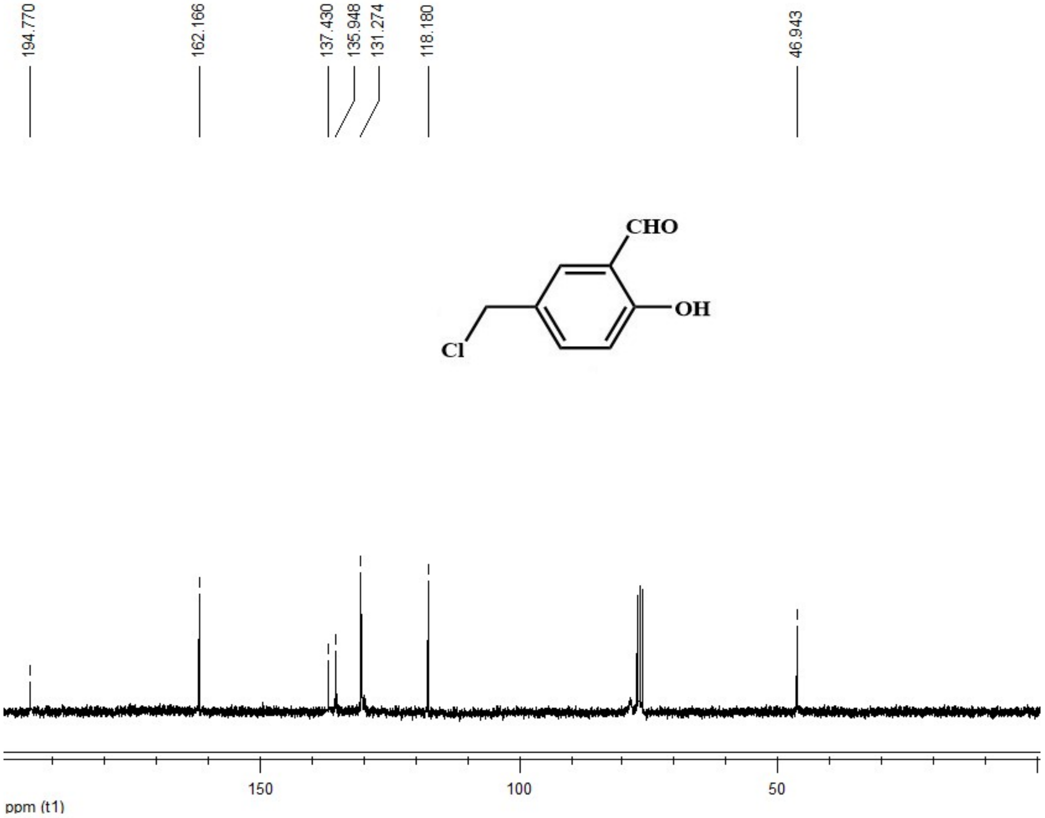

Fig. S4 ^{13}C-NMR spectrum of 5-chloromethyl salicylaldehyde (**1**) ($CDCl_3$, 75 MHz)

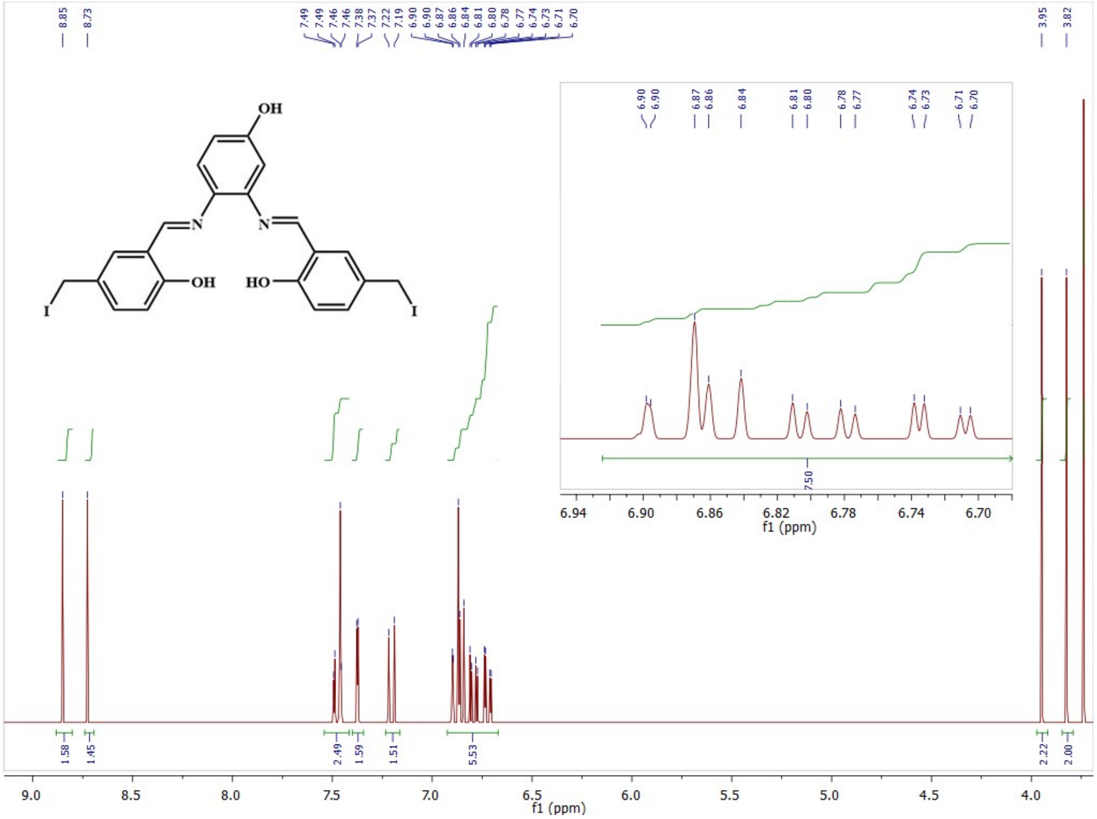

Fig. S5 ^{1}H-NMR spectrum of salen ligand (**3**) (DMSO-d_6, 300 MHz)

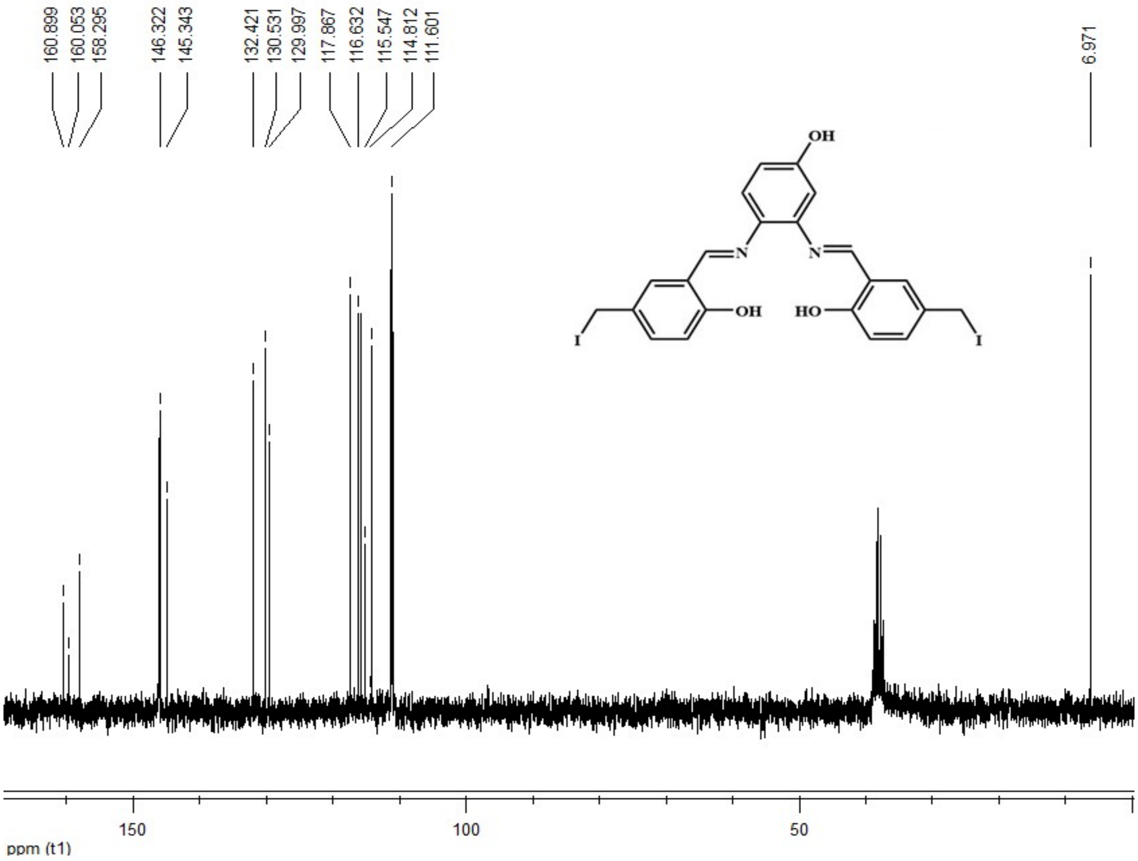

Fig. S6 ^{13}C-NMR spectrum of salen ligand (**3**) (DMSO-d_6, 75 MHz)

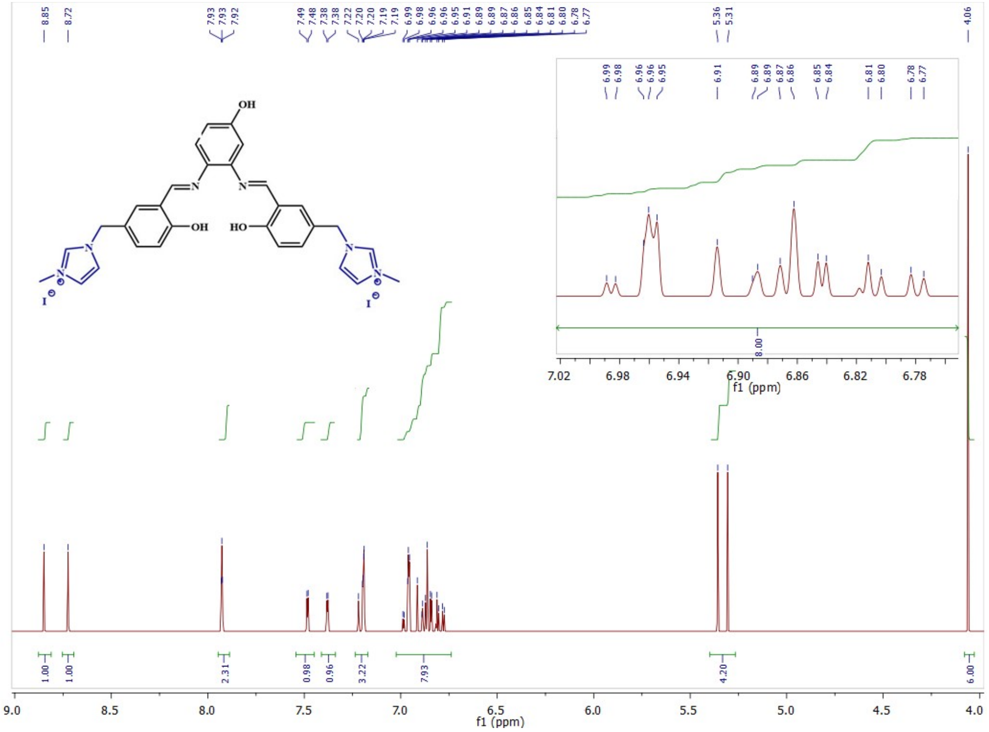

Fig. S7 ^{1}H-NMR spectrum of salen-[IM]I complex (**4**) (DMSO-d_6, 300 MHz)

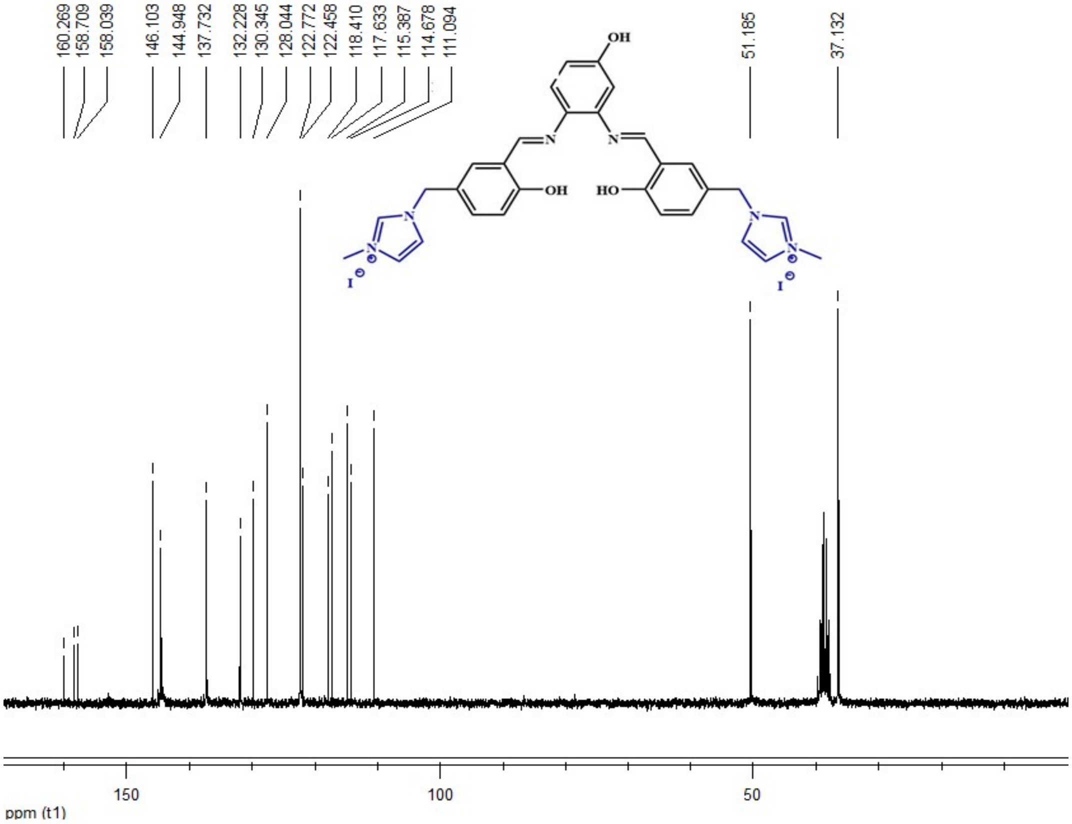

Fig. S8 ^{13}C-NMR spectrum of salen-[IM]I complex (**4**) (DMSO-d_6, 75 MHz)

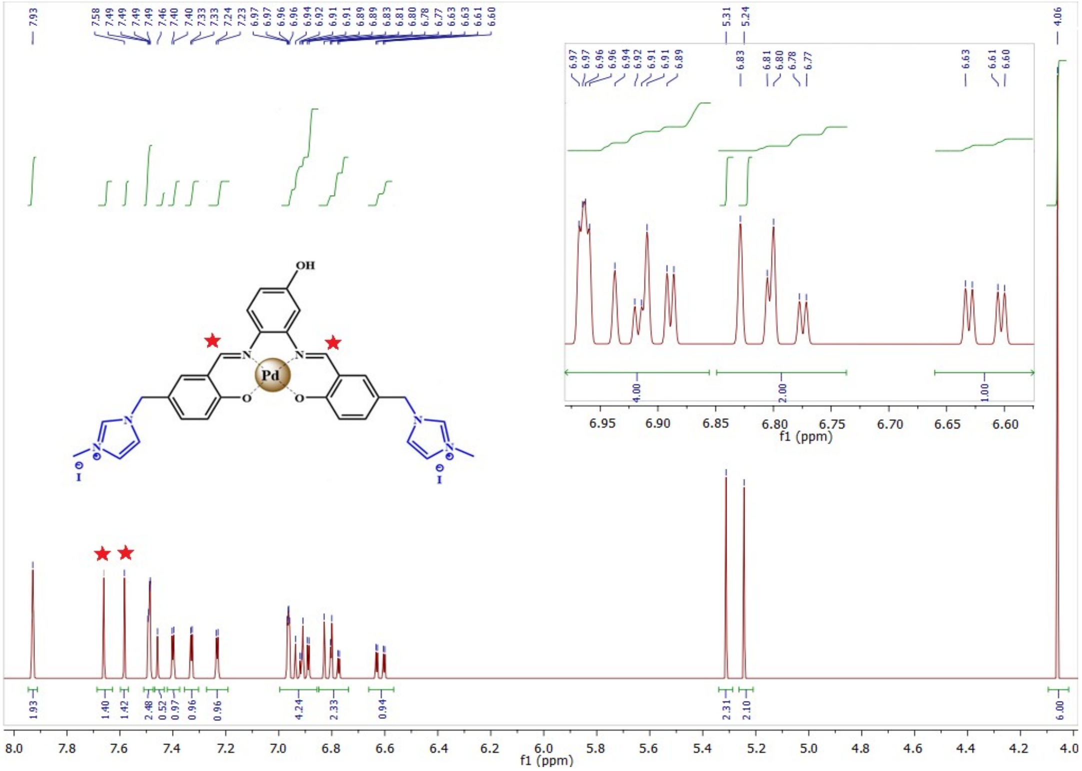

Fig. S9 ^{1}H-NMR spectrum of Pd(II)-salen-[IM]I complex (**5**) (DMSO-d_6, 300 MHz)

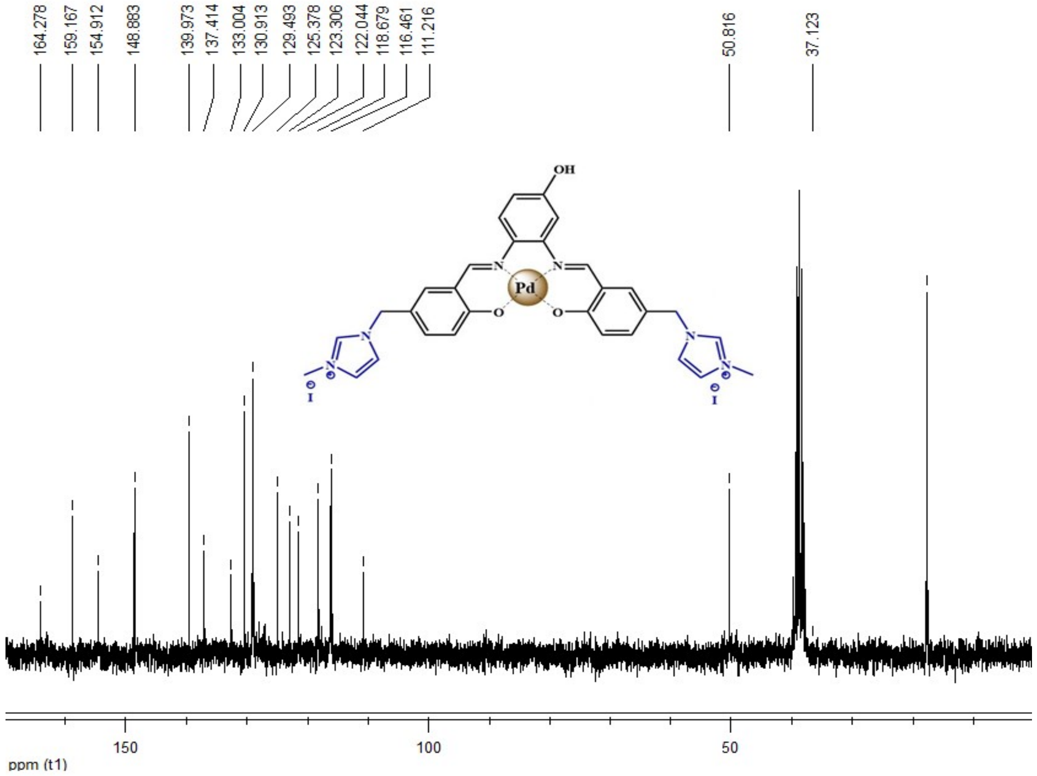

Fig. S10 ^{13}C-NMR spectrum of Pd(II)-salen-[IM]I complex (**5**) (DMSO-d_6, 75 MHz)

Table S1 Influence of N_2 inlet pressure over the contact time of the reaction mixture of iodobenzene with phenylacetylene with the FP@Si-Pd^{II}-Salen-[IM]OH[a]

Entry	N_2 inlet pressure (bar)	Time (min)[b]	Yield

			(%)[c]
1	0.0	10	30
2	0.1	40	70
3	0.3	70	96
4	0.5	180	96
5	0.7	260	95

[a] Reaction conditions: Iodobenzene (1.0 mmol), phenylacetylene (1.0 mmol), EtOH: H_2O (2:1, v/v, 5.0 mL), 50 °C (on a water bath), N_2 inlet, catalytic filter paper (**7**, placed on a glass funnel, containing 1.0 mmol Pd/95cm^2).
[b] Total time spent for 5 consecutive re-filtrations of the residue.
[c] Isolated yield.

Table S2 Effect of different Pd loading amounts on the catalytic paper **7** over the Sonogashira model reaction[a]

Entry	mmol[b] of Pd/95 cm^2	Time (min)[c]	Yield (%)[d]
1	0.2	70	72
2	0.5	70	86
3	1.0	70	96
4	1.5	78	96
5	2.0	100	92

[a] Reaction conditions: Iodobenzene (1.0 mmol), phenylacetylene (1.0 mmol), EtOH: H_2O (2:1, v/v, 5.0 mL), 50 °C (on a water bath), N_2 inlet (0.3 bar), catalytic filter paper (**7**, placed on a glass funnel)
[b] Based on ICP analysis
[c] Total time spent for 5 consecutive re-filtrations of the residue.
[d] Isolated yield.

Table S3 Screening of various solvent types over the efficiency of Sonogashira model reaction

Entry	Solvent	Time (min)	Yield (%)
1	H_2O: EtOH (1:2, v/v)	70	96
2	DMF	57	90
3	CH_3CN	65	80
4	H_2O	75	40
5	EtOH	65	85
6	MeOH	65	85
7	DMSO	95	95
8	Butyl acetate	110	55
9	Hexane	55	-

[a] Reaction conditions: Iodobenzene (1.0 mmol), phenylacetylene (1.0 mmol), solvent (5.0 mL), 50 °C (on a water bath), N_2 inlet (0.3 bar), catalytic filter paper (**7**, placed on a glass funnel, containing 1.0 mmol Pd/95cm^2).
[b] Based on ICP analysis
[c] Total time spent for 5 consecutive re-filtrations of the residue.
[d] Isolated yield.

Table S4 Influence of temperature over the efficiency of Sonogashira model reaction

Entry	T (°C)	Time (min)	Yield (%)
1	27	70	85
2	50	70	96
3	70	70	96

[a] Reaction conditions: Iodobenzene (1.0 mmol), phenylacetylene (1.0 mmol), EtOH: H_2O (2:1, v/v, 5.0 mL), T °C (on a water bath), N_2 inlet (0.3 bar), catalytic filter paper (**7**, placed on a glass funnel, containing 1.0 mmol Pd/95cm^2).
[b] Based on the ICP analysis.

[c] Total time spent for 5 consecutive re-filtrations of the residue.
[d] Isolated yield.

Table S5 Influence of area (in cm^2) over the efficiency of Sonogashira model reaction[a]

Entry	Area of FP@Si-Pd^{II}-Salen-[IM]OH (cm^2)	Time (min)[b]	Yield (%)[c]
1	1	70	N.R.
2	10	70	N.R.
3	20	70	35
4	40	70	65
5	80	70	77
6	95	70	80
7	120	70	80

[a] Reaction conditions: Iodobenzene (1.0 mmol), phenylacetylene (1.0 mmol), EtOH: H_2O (2:1, v/v, 5.0 mL), T °C (on a water bath), N_2 inlet (0.3 bar), a piece of catalytic filter paper (added in the mixture). The reaction was performed as a heterogamous catalytic reaction. [b] Total time spent for 5 consecutive re-filtrations of the residue. [c] Isolated yield.

ANOVA for Quadratic model

Table S6 ANOVA for Response Surface Quadratic Model in model Sonogashira cross-coupling reaction [analysis of variance table][a]

Source	Sum of Squares	df	Mean Square	F-value	p-value	
Block	132.49	2	66.25			
Model	7434.42	14	531.03	105.68	< 0.0001	significant
A-G.P	1426.04	1	1426.04	283.79	< 0.0001	
B-pd Content	1717.04	1	1717.04	341.69	< 0.0001	
C-Tem.	442.04	1	442.04	87.97	< 0.0001	
D-S.A.	425.04	1	425.04	84.58	< 0.0001	
AB	68.06	1	68.06	13.54	0.0028	
AC	7.56	1	7.56	1.50	0.2417	
AD	33.06	1	33.06	6.58	0.0235	
BC	0.0625	1	0.0625	0.0124	0.9129	
BD	52.56	1	52.56	10.46	0.0065	
CD	45.56	1	45.56	9.07	0.0100	
A^2	724.53	1	724.53	144.18	< 0.0001	
B^2	2750.86	1	2750.86	547.43	< 0.0001	
C^2	43.86	1	43.86	8.73	0.0112	
D^2	1.92	1	1.92	0.3821	0.5472	
Residual	65.33	13	5.03			
Lack of Fit	63.05	10	6.30	8.30	0.0541	not significant
Pure Error	2.28	3	0.7600			
Cor Total	7632.23	29				

[a] Factor coding is Coded. Sum of squares is Type III – Partial

The Model F-value of 105.68 implies the model is significant. There is only a 0.01% chance that an F-value this large could occur due to noise.
P-values less than 0.0500 indicate model terms are significant. In this case A, B, C, D, AB, AD, BD, CD, A^2, B^2, C^2 are significant model terms. Values greater than 0.1000 indicate the model terms are not significant. If there are many insignificant model terms (not counting those required to support hierarchy), model reduction may improve your model.

The Lack of Fit F-value of 8.30 implies there is a 5.41% chance that a Lack of Fit F-value this large could occur due to noise. Lack of fit is bad. This relatively low probability (<10%) is troubling.

Table S7 Fit Statistics of the results[a]

Std. Dev.	2.24	R^2	0.9913
Mean	78.25	Adjusted R^2	0.9819
C.V. %	2.86	Predicted R^2	0.9324
		Adeq Precision	38.9746

[a] The Predicted R^2 of 0.9324 is in reasonable agreement with the Adjusted R^2 of 0.9819; i.e. the difference is less than 0.2.
Adeq Precision measures the signal to noise ratio. A ratio greater than 4 is desirable. The ratio of 38.975 indicates an adequate signal. This model can be used to navigate the design space.

Table S8 Coefficients in Terms of Coded Factors[a]

Factor	Coefficient Estimate	df	Standard Error	95% CI Low	95% CI High	VIF
Intercept	91.60	1	0.9152	89.62	93.58	
Block 1	2.15	2				
Block 2	0.7067					
Block 3	-2.85					
A-G.P	7.71	1	0.4576	6.72	8.70	1.0000
B-pd Content	8.46	1	0.4576	7.47	9.45	1.0000
C-Tem.	4.29	1	0.4576	3.30	5.28	1.0000
D-S.A.	4.21	1	0.4576	3.22	5.20	1.0000
AB	-2.06	1	0.5604	-3.27	-0.8518	1.0000
AC	0.6875	1	0.5604	-0.5232	1.90	1.0000
AD	-1.44	1	0.5604	-2.65	-0.2268	1.0000
BC	-0.0625	1	0.5604	-1.27	1.15	1.0000
BD	1.81	1	0.5604	0.6018	3.02	1.0000
CD	-1.69	1	0.5604	-2.90	-0.4768	1.0000
A^2	-5.14	1	0.4280	-6.06	-4.21	1.05
B^2	-10.01	1	0.4280	-10.94	-9.09	1.05
C^2	-1.26	1	0.4280	-2.19	-0.3399	1.05
D^2	-0.2646	1	0.4280	-1.19	0.6601	1.05

[a] The coefficient estimate represents the expected change in response per unit change in factor value when all remaining factors are held constant. The intercept in an orthogonal design is the overall average response of all the runs. The coefficients are adjustments around that average based on the factor settings. When the factors are orthogonal the VIFs are 1; VIFs greater than 1 indicate multi-colinearity, the higher the VIF the more severe the correlation of factors. As a rough rule, VIFs less than 10 are tolerable.

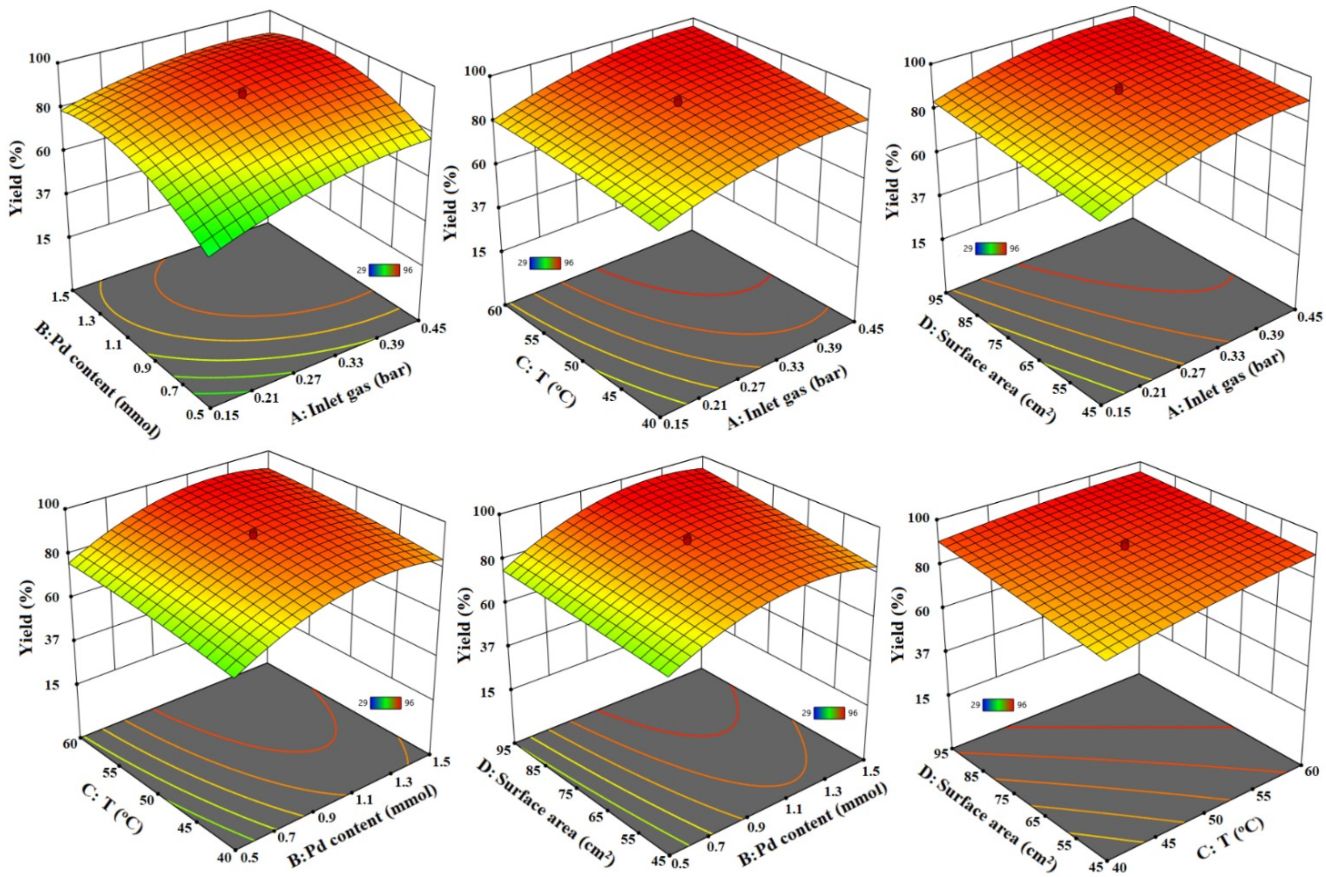

Fig. S11 The 3D central design curves for foundation of optimization ranges for maximum response in model Sonogashira reaction

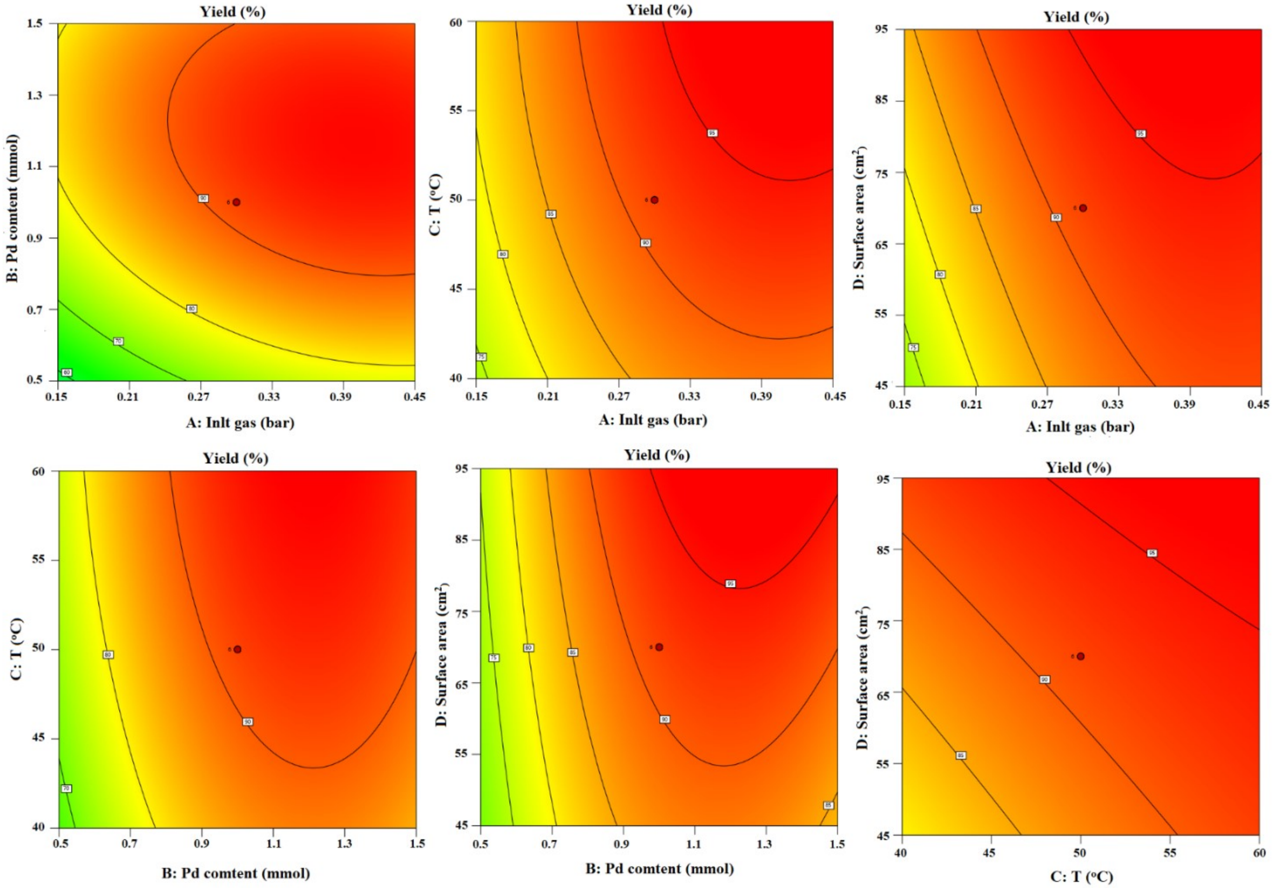

Fig. S12 The 2D central design curves for foundation of optimization ranges for maximum response in model Sonogashira reaction

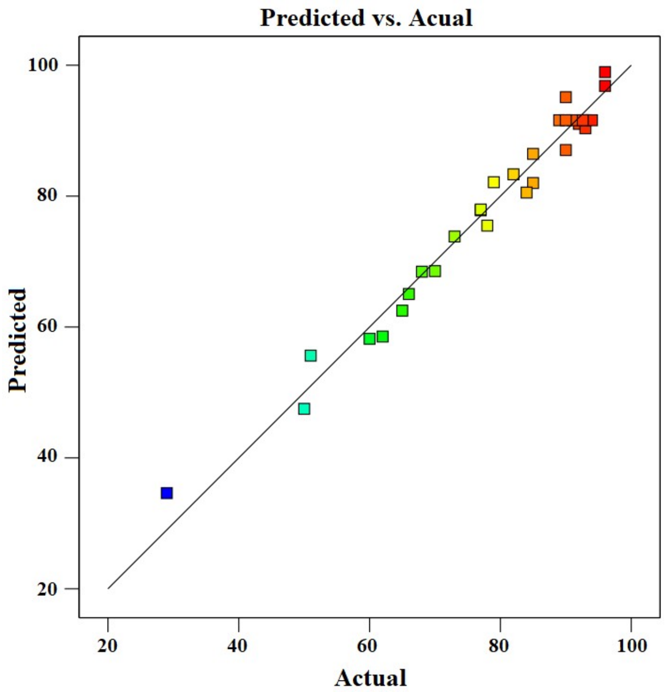

Fig. S13 Accuracy of the predicted model *vs*. actual values

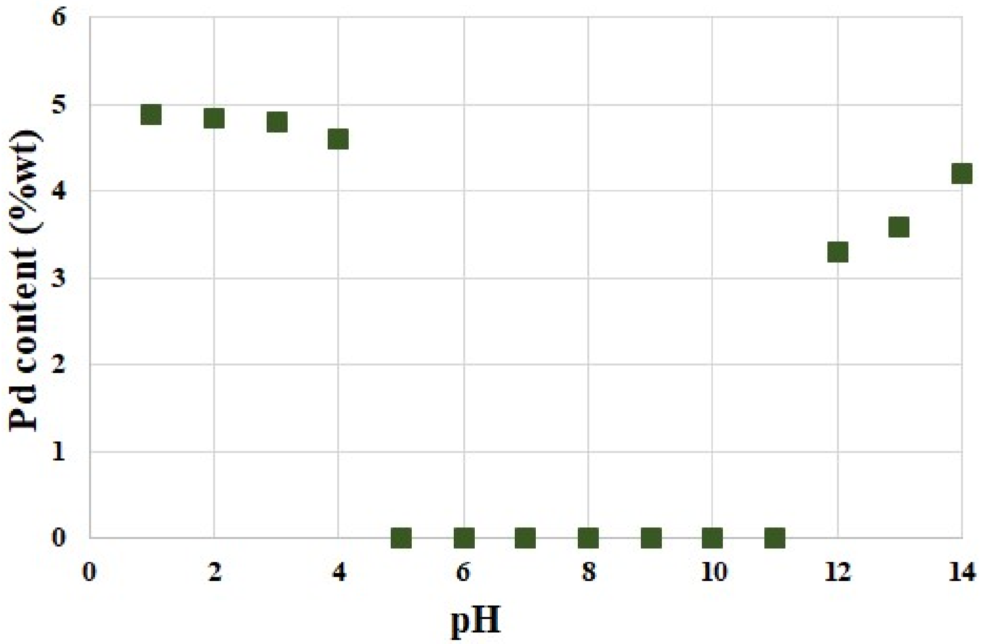

Fig. S14 Influence of pH over Pd leaching of FP@Si-Pd^{II}-Salen-[IM]OH

Table S9. Comparison of the catalytic activity of FP@Si-Pd^{II}-Salen-[IM]OH with the previously reported Pd-based heterogeneous catalyst for the Sonogashira cross-coupling preparation of **10a**

Cat.	Solid support	Conditions	Cat. recycling	Metal leaching	*t.* (min)	C. (%)	TOF (h^{-1})	[Ref.]
Pd@MGO-D-NH_2	GO[a]	K_2CO_3, H_2O:EtOH, reflux	Magnet	Yes	60	95[b]	112	[2]
PS anchored $Pd^{(II)}$ azo complex	PS[c]	K_2CO_3, H_2O, 70 °C	Filtration	No	360	100	34	[3]
Pd/C	AC[d]	DES,[e] Et_3N, 60 °C	Extraction	N.A.	180	90[b,f]	15	[4]
PdMgAl-LDH-1[g]	LDH	K_2CO_3, NaVc,[h] CTAB,[i] H_2O, 80 °C	Filtration	Yes	60	94[b]	470	[5]
Pd-NHC-MIL-101 (Cr)	MIL-101(Cr)[j]	DMF, K_2CO_3, 110 °C	Centrifuge	Yes	8	92[b]	12	[6]
Pd@Fe_3O_4/AMOCAA[k]	Fe_3O_4	K_2CO_3, EtOH: H_2O, 80 °C	Magnet	Yes	60	96[b]	24	[7]
FP@Si-Pd^{II}-Salen-[IM]OH	Cellulose paper	EtOH: H_2O, 50 °C, N_2 atm.	Simple washing	No	70	96		This work

a Graphene oxide. [b] Yield. [c] Polystyrene. [d] Activated carbon. [e] Deep eutectic solvent. [f] For 1-iodo-4-nitrobenzene, [g] Mg-Al layered double hydroxide. [h] Sodium ascorbate. [i] TBAB=tetrabutylammonium bromide. [j] MIL=Matérial Institut Lavoisier; a chromium terephthalate metal–organic framework. [k] AMOCAA = 2-(7-amino-4-methyl-2-oxo-2H-chromen-3-yl)acetic acid.

References

[1] M. Kazemnejadi, S.A. Alsvi, Z. Rezazadeh, M.A. Nasseri, A. Allahresani and M. Esmaeilpour, *Green Chem.*, 2019, **21**, 1718-1734.

[2] Tarahomi, M., Alinezhad, H., & Maleki, B. (2019). Immobilizing Pd nanoparticles on the ternary hybrid system of graphene oxide, Fe3O4 nanoparticles, and PAMAM dendrimer as an efficient

support for catalyzing sonogashira coupling reaction. *Applied Organometallic Chemistry*, *33*(11), e5203.

[3] Islam, S. M., Mondal, P., Roy, A. S., Mondal, S., & Hossain, D. (2010). Heterogeneous Suzuki and copper-free Sonogashira cross-coupling reactions catalyzed by a reusable palladium (II) complex in water medium. *Tetrahedron Letters*, *51*(15), 2067-2070.

[4] Messa, F., Dilauro, G., Perna, F. M., Vitale, P., Capriati, V., & Salomone, A. (2020). Sustainable ligand-free heterogeneous palladium-catalyzed sonogashira cross-coupling reaction in deep eutectic solvents. *ChemCatChem*, *12*(7), 1979-1984.

[5] Zhang, S. Y., Yu, K., Guo, Y. S., Mou, R. Q., Lu, X. F., & Guo, D. S. (2018). Preparation and Reactivation of Heterogeneous Palladium Catalysts and Applications in Sonogashira, Suzuki, and Heck Reactions in Aqueous Media. *ChemistryOpen*, *7*(10), 803-813.

[6] Niknam, E., Panahi, F., & Khalafi-Nezhad, A. (2021). Immobilized Pd on a NHC-functionalized metal-organic FrameworkMIL-101 (Cr): An efficient heterogeneous catalyst in the heck and copper-free Sonogashira coupling reactions. *Journal of Organometallic Chemistry*, *935*, 121676.

[7] Tamoradi, T., Daraie, M., & Heravi, M. M. (2020). Synthesis of palladated magnetic nanoparticle (Pd@ Fe3O4/AMOCAA) as an efficient and heterogeneous catalyst for promoting Suzuki and Sonogashira cross-coupling reactions. *Applied Organometallic Chemistry*, *34*(4), e5538.
